# The Synthesis and Biological Evaluation of Aloe-Emodin-Coumarin Hybrids as Potential Antitumor Agents

**DOI:** 10.3390/molecules27196153

**Published:** 2022-09-20

**Authors:** Hai Shang, Yue Hu, Jingrong Li, Lingyu Li, Yu Tian, Xiaoxue Li, Qi Wu, Zhongmei Zou

**Affiliations:** 1 Institute of Medicinal Plant Development, Chinese Academy of Medical Sciences and Peking Union Medical College, Beijing 100193, China; 2 School of Basic Medical Sciences, Guizhou Medical University, Guiyang 550025, China; 3 School of Traditional Chinese Materia Medica, Shenyang Pharmaceutical University, Shenyang 110016, China; 4 School of Pharmaceutical Engineering, Shenyang Pharmaceutical University, Shenyang 110016, China

**Keywords:** aloe-emodin, coumarin, hybrid, structural modification, antitumor activity

## Abstract

A series of novel aloe-emodin–coumarin hybrids were designed and synthesized. The antitumor activity of these derivatives was evaluated against five human tumor cell lines (A549, SGC-7901, HepG2, MCF-7 and HCT-8). Some of the synthesized compounds exhibited moderate to good activity against one or more cell lines. Particularly, compound **5d** exhibited more potent antiproliferative activity than the reference drug etoposide against all tested tumor cell lines, indicating that it had a broad spectrum of antitumor activity and that it may provide a promising lead compound for further development as an antitumor agent by structural modification. Furthermore, the structure–activity relationship study of the synthesized compounds was also performed.

## 1. Introduction

**Figure 1:**
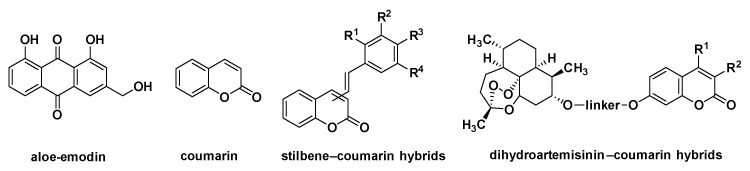
Structures of aloe-emodin, coumarin and the hybrid derivatives of coumarin.

Aloe-emodin (1,8-dihydroxy-3-hydroxymethyl-anthraquinone), a representative natural anthraquinone compound, is isolated from some traditional medicinal plants such as *Rheum palmatum* L. and *Aloe vera* L (Figure 1). Modern pharmacological studies have shown that aloe-emodin exhibits a broad range of bioactivity profiles, which include antitumor [1,2,3,4,5], anti-inflammatory [6,7], antiviral [8], antimicrobial [9] and antifibrosis effects [10]. It is noteworthy that its antitumor activity has attracted special attention. Some studies have shown that aloe-emodin exerts the anti-tumor effect through inhibiting proliferation and inducing the apoptosis of certain cancer cells [11,12,13,14,15]. However, aloe-emodin itself is not sufficient to apply as an anti-tumor drug, and the development of its derivatives to improve its therapeutic efficacies is necessary. 

Coumarin represents an important class of heterocyclic skeletons, which is widely distributed in diverse natural products. Coumarin-based natural and synthetic derivatives exhibit a wide range of pharmacological properties [16,17,18,19], and antitumor activity is one of the most important pharmacological activities among them [20,21,22]. Biological investigations have revealed that coumarin derivatives can exert antitumor efficiency through binding to various biological targets such as sulfatase [23], aromatase [24] and protein kinase [25]. Owing to its privileged structure and impressive antitumor activity, coumarin is often introduced into many natural and synthetic compounds in the design of antitumor drugs [26,27]. For example, Belluti et al. adopted a molecular hybridization strategy to produce stilbene-coumarin hybrids, and the hybrid compounds showed higher antitumor activities than the reference compound resveratrol [28]. Guo and co-workers synthesized a series of dihydroartemisinin-coumarin hybrids as potential antitumor agents, and the evaluation of their antitumor activity also proved that the synthetic compounds had great antitumor activity against the MDA-MB-231 and HT-29 cell lines [29]. The above research indicates that the hybridization of coumarin with other antitumor compounds is an effective strategy to search for new antitumor agents, and the resulting hybrid compounds often have more potent antitumor activity than the parent compound. These stimulated us to design and synthesize the hybrids of aloe-emodin and coumarin as antitumor agents by a molecular hybridization strategy to improve the affinity and efficacy of aloe-emodin.

Herein, a series of aloe-emodin-coumarin hybrids were synthesized, and their antiproliferative activity was evaluated against a panel of human tumor cell lines using etoposide as a reference. The structure-activity relationship of the derivatives is also discussed.

## 2. Results and Discussion

### 2.1. Chemistry

**Scheme 1:**
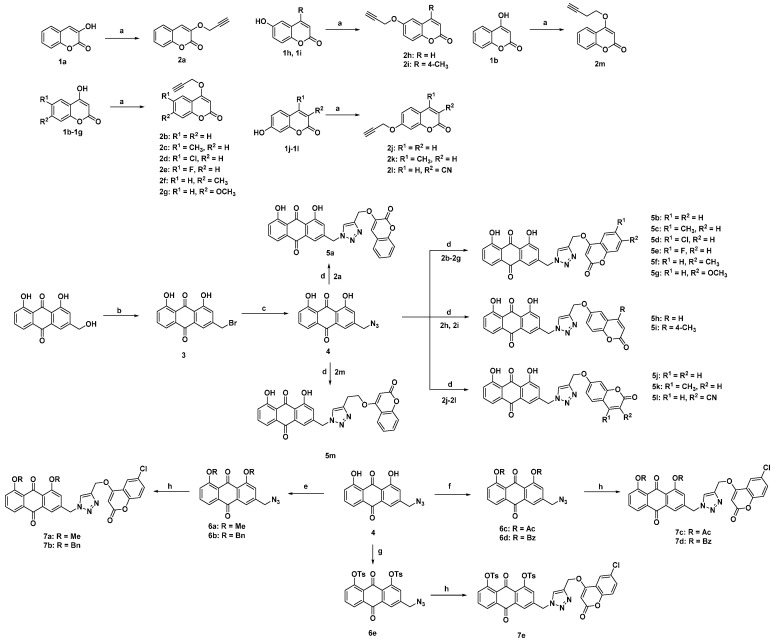
Reagents and conditions: (a) K_2_CO_3_, 3-bromoprop-1-yne (for **2a**–**l**) or 4-bromobut-1-yne (for **2m**), DMF, 60 °C, 2 h; (b) PPh_3_, CBr_4_, THF, room temperature, 6 h; (c) NaN_3_, CH_3_CN, 60 °C, 3 h; (d) **2a**–**m**, copper(I) thiophene-2-carboxylate, CH_2_Cl_2_, room temperature, 6 h; (e) K_2_CO_3_, methyl iodide (for **6a**) or benzyl bromide (for **6b**), DMF, 60 °C, 4 h; (f) acetyl chloride (for **6c**) or benzoyl chloride (for **6d**), DMAP, DIPEA, CH_2_Cl_2_, room temperature, 4 h; (g) K_2_CO_3_, TsCl, CH_3_COCH_3_, 60 °C, 5 h; (h) **2d**, copper(I) thiophene-2-carboxylate, CH_2_Cl_2_, room temperature, 6 h.

The synthetic strategy employed for the synthesis of aloe-emodin–coumarin derivatives is depicted in Scheme 1. *O*-propargyl and -butynyl coumarin derivatives **2a**–**m** were obtained in a single step by the reaction of corresponding hydroxycoumarin with propargyl bromide or 4-bromobut-1-yne in the presence of K_2_CO_3_ in DMF. The treatment of commercially available aloe-emodin with CBr_4_ and PPh_3_ in tetrahydrofran afforded bromide **3** in 94% yields [30]. Then, the intermediate bromide **3** was reacted with NaN_3_ through nucleophilic substitution to provide azido derivative **4**, which was finally subjected to the click reaction with *O*-propargyl or -butynyl coumarin derivatives (**2a**–**m**) in the presence of copper(I) thiophene-2-carboxylate in dichloromethane to afford the target products (**5a**–**m**) [31]. To further investigate the structure-activity relationship of 1- and 8-disubstitution on the anthraquinone core of compound **5d** (the most active compound among derivatives **5a**–**m**), a series of **5d** analogs **7a**–**e** were synthesized. The methylation or benzylation of **4** with methyl iodide or benzyl bromide in the presence of K_2_CO_3_ was performed to give dimethylated or dibenzylated intermediates **6a** and **6b**, respectively. Diacetylated intermediate **6c** and dibenzoylated intermediate **6d** were obtained by the acylation of **4**. The sulfonylation of **4** with *p*-toluenesulfonyl chloride gave disulfonylated intermediate **6e**. Finally, **6a**–**e** reacted with **2d** under the catalysis of copper(I) thiophene-2-carboxylate to give **7a**–**e**. The structures of the target compounds are shown in Table 1 and were identified by HRMS, ^1^H-NMR and ^13^C-NMR spectral analysis (^1^H-NMR and ^13^C-NMR spectra of the synthesized compounds are available in the Supplementary Materials).

### 2.2. Biological Results and Discussion

All of the target compounds were evaluated for their antiproliferative activities against five cancer cell lines (A549, SGC-7901, HepG2, MCF-7 and HCT-8) with etoposide as a positive reference using the MTT assay. To determine the selectivity, the cytotoxic activities of several compounds with good antitumor activities were also evaluated against the Hk-2 normal cell line (Human Kidney-2). The results are summarized in Table 2.

Compared to the parent compound aloe-emodin, some compounds displayed promising antiproliferative activity against one or more cell lines. Among them, compound **5d** exhibited the most potent antiproliferative activity against all the tested cell lines. It showed 3–9-fold increased activity compared to the positive control, etoposide. In addition, **5f** also exhibited strong potency against all five cell lines, which was comparable or superior to that of etoposide. It is worth noting that five synthesized compounds (**5a**, **5d**, **5f**, **5g** and **5j**) exhibited moderate to good activities against all the tested cell lines, which indicated that these derivatives possessed a broad spectrum of antitumor activity. The results of the cytotoxic activities of **5a**, **5d**, **5f**, **5g** and **7c** against the Hk-2 cell line showed that the synthetic compounds also displayed similar cytotoxic activity compared with the tested tumor cell lines, which did not show obvious selectivity. 

The preliminary structure–activity relationship (SAR) study indicated that the linking position of coumarin moiety had an effect on their antiproliferative activity. The derivatives linked at the 3-, 4- and 7-position of coumarin were more active than others linked at the 6-position. In the same series of derivatives (containing the same linking position of coumarin), both the position and electron donor–acceptor properties of the substituents on coumarin significantly affected the activity. For the derivatives linked at the 4-position of coumarin, the chloro group in the 5-position of coumarin (**5d**) showed better activity than others, and the derivatives bearing a methyl (**5f**) or methoxy (**5g**) group in the 6-position of coumarin showed slightly weaker activity than **5d**. It was noted that the replacement of the chloro with the fluoro group (**5e**) in the 5-position of coumarin resulted in the loss of activity. Regarding the derivatives linked at the 6-position of coumarin, the substituent at the 3-position of coumarin (**5k**) was better than that at the 4-position (**5l**). Moreover, to investigate the influence of the linker length to activity, **5m** with an extended linker length was then synthesized, and the activity of it showed equal to or slightly less than that of **5a** against four tumor cell lines (SGC-7901, HepG2, MCF-7 and HCT-8). The results indicated that extending the linker length had no significant effect on activity.

The effect of the substituent at the 1- and 8-position on an anthraquinone core was also investigated. The results suggested that the substituent size and steric properties had a remarkable effect on their antiproliferative activity. 1, 8-diacetyl-substituted derivative **7c** displayed similar or slightly decreased potency to 1, 8-unsubstituted derivative **5d** against four tumor cell lines (SGC-7901, HepG2, MCF-7 and HCT-8). However, after replacement of the acetyl group with the bulky benzoyl and benzenesulfonyl moieties, compounds **7d** and **7e** exhibited weak or no activity. A similar phenomenon could also be found by the replacement of the methyl (**7a**) group with the benzyl (**7b**). The results suggested that the introduction of the bulky substituents at the 1- and 8-position on anthraquinone was detrimental for activity.

## 3. Materials and Methods

### 3.1. General Information

NMR spectra were recorded on a Bruker AVANCE III 600 or a Bruker AVANCE III 500 NMR spectrometer instrument (Bruker, Rheinstetten, Germany). Solvent signals (DMSO-*d*_6_: *δ*_H_ = 2.50 ppm/*δ*_C_ = 39.52 ppm) were used as references. High-resolution mass spectra (HRMS) were recorded on a Waters SYNAPT G2 HDMS (Waters, Milford, MA, USA). The reactions were monitored by thin-layer chromatography on plates (GF_254_) supplied by Yantai Chemicals (Yantai, China). Silica gel column chromatography was performed using 200–300 mesh silica gel supplied by Tsingtao Haiyang Chemicals (Tsingtao, China). Unless otherwise noted, all common reagents and solvents were obtained from commercial suppliers (Beijing InnoChem Science & Technology Co., Ltd., Beijing, China) without further purification. 

### 3.2. Chemistry

#### 3.2.1. General Procedures for O-Propargyl and O-Butynyl Coumarin Derivatives (**2a**–**m**)

3-bromoprop-1-yne or 4-bromobut-1-yne (2.0 mmol) and anhydrous K_2_CO_3_ (1.5 mmol) were added to a solution of the corresponding hydroxycoumarin (1.0 mmol) in DMF (5 mL). The reaction was heated to 60 °C for 2 h. Upon completion, the reaction was cooled down to room temperature, and then the reaction mixture was diluted with water and extracted three times with ethyl acetate. The organic layers were dried with dry Na_2_SO_4_, they were filtered, and the filtrate was removed under reduced pressure. The residue was purified by column chromatography to give compounds **2a**–**m** (Yield: 40~96%).

#### 3.2.2. General Procedure for the Synthesis of 3-(Bromomethyl)-1,8-Dihydroxyanthracene-9,10-Dione (**3**)

CBr_4_ (15.34 g, 46.25 mmol) was added portion-wise to a solution of PPh_3_ (12.13 g, 46.25 mmol) in THF (50 mL) at room temperature. The mixture was left stirring for 10 min, and then aloe-emodin (5.0 g, 18.5 mmol) was added. After being stirred at room temperature for 6 h, the reaction mixture was filtered, and the filtrate was evaporated to give a residue that was subjected to column chromatography (petroleum ether:CH_2_Cl_2_ = 1:1) to obtain compound **3** (5.83 g, 94%). ^1^H-NMR (600 MHz, DMSO-*d*_6_) *δ*: 11.92 (s, 1H, OH-8), 11.91 (s, 1H, OH-1), 7.82 (dd, *J* = 8.3 Hz, 7.5 Hz, 1H, H-6), 7.78 (d, *J* = 1.7 Hz, 1H, H-4), 7.72 (dd, *J* = 7.5 Hz, 1.1 Hz, 1H, H-5), 7.47 (d, *J* = 1.7 Hz, 1H, H-2), 7.40 (dd, *J* = 8.3 Hz, 1.1 Hz, 1H, H-7), 4.82 (s, 2H, H-11).

#### 3.2.3. General Procedure for the Synthesis of 3-(Azidomethyl)-1,8-Dihydroxyanthracene-9,10-Dione (**4**)

NaN_3_ (117 mg, 1.8 mmol) was added to a solution of compound **3** (500 mg, 1.5 mmol) in dry CH_3_CN (10 mL) at room temperature. The reaction mixture was heated to 60 °C for 3 h. Upon completion, the reaction was cooled down to room temperature, and then the reaction mixture was diluted with water and extracted three times with CH_2_Cl_2_. The organic layers were dried with dry Na_2_SO_4_, they were filtered, and the filtrate was removed under reduced pressure. The residue was purified by column chromatography (petroleum ether:CH_2_Cl_2_ = 1:1) to give compound **4** (375 mg, 85%). ^1^H-NMR (600 MHz, DMSO-*d*_6_) *δ*: 11.92 (s, 1H, OH-8), 11.91 (s, 1H, OH-1), 7.81 (dd, *J* = 8.4 Hz, 7.4 Hz, 1H, H-6), 7.71 (dd, *J* = 7.4 Hz, 1.1 Hz, 1H, H-5), 7.68 (d, *J* = 1.6 Hz, 1H, H-4), 7.39 (dd, *J* = 8.4 Hz, 1.1 Hz, 1H, H-7), 7.36 (d, *J* =1.6 Hz, 1H, H-2), 4.68 (s, 2H, H-11).

#### 3.2.4. General Procedures for the Synthesis of Aloe-Emodin–Coumarin Hybrids (**5**)

Compound **2** (0.24 mmol) and copper(I) thiophene-2-carboxylate (0.08 mmol) were added to a solution of compound **4** (0.2 mmol) in CH_2_Cl_2_ (10 mL). After being stirred at room temperature for 6 h, the reaction mixture was concentrated, and the residue was purified by silica gel column chromatography to give compound **5**.

*1,8-dihydroxy-3-((4-(((2-oxo-2H-chromen-3-yl)oxy)methyl)-1H-1,2,3-triazol-1-yl)methyl)anthracene-9,10-dione* (**5a**) yellow solid. Yield: 57%; mp: 252.3–254.1 °C; ^1^H-NMR (500 MHz, DMSO-*d_6_*) *δ* 11.91–11.89 (m, 2H, OH-1, OH-8), 8.49 (s, 1H, H-12), 7.81 (dd, *J* = 8.4 Hz, 7.4 Hz, 1H, H-6), 7.71 (dd, *J* = 7.4 Hz, 0.9 Hz, 1H, H-5), 7.62–7.59 (m, 2H, H-4, H-5′), 7.57 (s, 1H, H-4′), 7.44 (ddd, *J* = 8.0 Hz, 7.4 Hz, 1.5 Hz, 1H, H-7′), 7.39 (dd, *J* = 8.4 Hz, 0.9 Hz, 1H, H-7), 7.36 (d, *J* = 8.0 Hz, 1H, H-8′), 7.32 (ddd, *J* = 7.6 Hz, 7.4 Hz, 0.9 Hz, 1H, H-6′), 7.28 (d, *J* = 1.4 Hz, 1H, H-2), 5.83 (s, 2H, H-11), 5.26 (s, 2H, H-14); ^13^C-NMR (125 MHz, DMSO-*d_6_*) *δ* 191.5, 181.2, 161.4, 161.3, 156.5, 149.2, 145.6, 142.5, 142.0, 137.5, 133.9, 133.3, 128.6, 127.0, 125.8, 124.8, 124.5, 122.9, 119.6, 119.4, 118.3, 116.0, 115.8, 115.7, 115.1, 62.1, 52.1; HRMS *m*/*z* calcd for C_27_H_18_N_3_O_7_ [M + H]^+^: 496.1145; found: 496.1147.

*1,8-dihydroxy-3-((4-(((2-oxo-2H-chromen-4-yl)oxy)methyl)-1H-1,2,3-triazol-1-yl)methyl)anthracene-9,10-dione* (**5b**) yellow solid. Yield: 46%; mp: 272.5–273.8 °C; ^1^H-NMR (500 MHz, DMSO-*d_6_*) *δ* 11.91 (s, 1H, OH-8), 11.90 (s, 1H, OH-1), 8.58 (s, 1H, H-12), 7.82 (dd, *J* = 8.4 Hz, 7.5 Hz, 1H, H-6), 7.76 (dd, *J* = 7.9 Hz, 1.5 Hz, 1H, H-5′), 7.72 (dd, *J* = 7.5 Hz, 0.9 Hz, 1H, H-5), 7.66 (ddd, *J* = 8.1 Hz, 7.4 Hz, 1.5 Hz, 1H, H-7′), 7.59 (d, *J* = 1.5 Hz, 1H, H-4), 7.41 (d, *J* = 8.1 Hz, 1H, H-8′), 7.40 (dd, *J* = 8.4 Hz, 0.9 Hz, 1H, H-7), 7.34 (ddd, *J* = 7.9 Hz, 7.4 Hz, 0.8 Hz, 1H, H-6′), 7.29 (d, *J* = 1.5 Hz, 1H, H-2), 6.16 (s, 1H, H-3′), 5.85 (s, 2H, H-11), 5.46 (s, 2H, H-14); ^13^C-NMR (150 MHz, DMSO-*d_6_*) *δ* 191.6, 181.3, 164.4, 161.6, 161.4, 161.4, 152.8, 145.6, 141.6, 137.5, 133.9, 133.3, 132.9, 126.1, 124.6, 124.3, 122.9, 119.5, 118.2, 116.5, 116.1, 115.8, 115.1, 91.4, 62.8, 52.1; HRMS *m*/*z* calcd for C_27_H_18_N_3_O_7_ [M + H]^+^: 496.1145; found: 496.1148.

*1,8-dihydroxy-3-((4-(((6-methyl-2-oxo-2H-chromen-4-yl)oxy)methyl)-1H-1,2,3-triazol-1-yl)methyl)anthracene-9,10-dione* (**5c**) yellow solid. Yield: 49%; mp: 274.9–276.9 °C; ^1^H-NMR (500 MHz, DMSO-*d_6_*) *δ* 11.91 (s, 1H, OH-8), 11.90 (s, 1H, OH-1), 8.57 (s, 1H, H-12), 7.82 (dd, *J* = 8.4 Hz, 7.5 Hz, 1H, H-6), 7.71 (dd, *J* = 7.5 Hz, 0.8 Hz, 1H, H-5), 7.58 (d, *J* = 1.5 Hz, 1H, H-4), 7.51 (d, *J* = 2.0 Hz, 1H, H-5′), 7.46 (dd, *J* = 8.5 Hz, 2.0 Hz, 1H, H-7′), 7.40 (dd, *J* = 8.4 Hz, 0.8 Hz, 1H, H-7), 7.32–7.27 (m, 2H, H-2, H-8′), 6.13 (s, 1H, H-3′), 5.85 (s, 2H, H-11), 5.45 (s, 2H, H-14), 2.34 (s, 3H, CH_3_-6′); ^13^C-NMR (125 MHz, DMSO-*d_6_*) *δ* 191.5, 181.3, 164.4, 161.7, 161.4, 161.4, 151.0, 145.6, 141.5, 137.5, 133.9, 133.7, 133.6, 133.3, 126.1, 124.6, 122.9, 122.3, 119.4, 118.1, 116.3, 116.0, 115.8, 114.8, 91.3, 62.7, 52.1, 20.3; HRMS *m*/*z* calcd for C_28_H_20_N_3_O_7_ [M + H]+: 510.1301; found: 510.1299.

*3-((4-(((6-chloro-2-oxo-2H-chromen-4-yl)oxy)methyl)-1H-1,2,3-triazol-1-yl)methyl)-1,8-dihydroxyanthracene-9,10-dione* (**5d**) yellow solid. Yield: 82%; mp: 286.3–288.1 °C; ^1^H-NMR (500 MHz, DMSO-*d_6_*) *δ* 11.92–11.89 (m, 2H, OH-1, OH-8), 8.59 (s, 1H, H-12), 7.82 (dd, *J* = 8.4 Hz, 7.5 Hz, 1H, H-6), 7.73–7.67 (m, 3H, H-5, H-5′, H-7′), 7.58 (d, *J* = 1.5 Hz, 1H, H-4), 7.47 (d, *J* = 8.7 Hz, 1H, H-8′), 7.40 (dd, *J* = 8.4 Hz, 1.0 Hz, 1H, H-7), 7.29 (d, *J* = 1.5 Hz, 1H, H-2), 6.24 (s, 1H, H-3′), 5.85 (s, 2H, H-11), 5.47 (s, 2H, H-14); ^13^C-NMR (150 MHz, DMSO-*d_6_*) *δ* 191.6, 181.2, 163.2, 161.4, 161.4, 161.1, 151.5, 145.6, 141.4, 137.5, 133.9, 133.3, 132.6, 128.3, 126.1, 124.6, 122.9, 122.0, 119.5, 118.8, 118.1, 116.6, 116.0, 115.8, 92.3, 63.1, 52.2; HRMS *m*/*z* calcd for C_27_H_17_ClN_3_O_7_ [M + H]^+^: 530.0755; found: 530.0759.

*3-((4-(((6-fluoro-2-oxo-2H-chromen-4-yl)oxy)methyl)-1H-1,2,3-triazol-1-yl)methyl)-1,8-dihydroxyanthracene-9,10-dione* (**5e**) yellow solid. Yield: 58%; mp: 245.9–247.8 °C; ^1^H-NMR (500 MHz, DMSO-*d_6_*) *δ* 11.94–11.80 (m, 2H, OH-1, OH-8), 8.60 (s, 1H, H-12), 7.79 (dd, *J* = 8.4 Hz, 7.4 Hz, 1H, H-6), 7.68 (dd, *J* = 7.4 Hz, 0.5 Hz, 1H, H-5), 7.55 (d, *J* = 1.3 Hz, 1H, H-4), 7.51 (dd, *J* = 8.3 Hz, 2.8 Hz, 1H, H-5′), 7.48–7.44 (m, 2H, H-7′, H-8′), 7.37 (dd, *J* = 8.4 Hz, 0.5 Hz, 1H, H-7), 7.27 (d, *J* = 1.3 Hz, 1H, H-2), 6.23 (s, 1H, H-3′), 5.84 (s, 2H, H-11), 5.47 (s, 2H, H-14); ^13^C-NMR (150 MHz, DMSO-*d_6_*) *δ* 191.5, 181.2, 163.5, 163.5, 161.4, 161.4 (d, *J* = 2.3 Hz), 158.1 (d, *J* = 241.3 Hz), 149.2, 145.6, 141.5, 137.5, 133.8, 133.3, 126.1, 124.6, 122.9, 120.3 (d, *J* = 24.6 Hz), 119.4, 118.8 (d, *J* = 8.5 Hz), 118.1, 116.2 (d, *J* = 9.3 Hz), 116.0, 115.8, 108.5 (d, *J* = 25.5 Hz), 92.2, 63.1, 52.2; HRMS *m*/*z* calcd for C_27_H_17_FN_3_O_7_ [M + H]^+^: 514.1051; found: 514.1053.

*1,8-dihydroxy-3-((4-(((7-methyl-2-oxo-2H-chromen-4-yl)oxy)methyl)-1H-1,2,3-triazol-1-yl)methyl)anthracene-9,10-dione* (**5f**) yellow solid. Yield: 85%; mp: 283.3–284.8 °C; ^1^H-NMR (600 MHz, DMSO-*d_6_*) *δ* 11.94–11.90 (m, 2H, OH-1, OH-8), 8.58 (s, 1H, H-12), 7.82 (dd, *J* = 8.4 Hz, 7.5 Hz, 1H, H-6), 7.72 (dd, *J* = 7.5 Hz, 0.8 Hz, 1H, H-5), 7.63 (d, *J* = 8.1 Hz, 1H, H-5′), 7.60 (d, *J* = 1.2 Hz, 1H, H-4), 7.40 (dd, *J* = 8.4 Hz, 0.8 Hz, 1H, H-7), 7.30 (d, *J* = 1.2 Hz, 1H, H-2), 7.24 (d, *J* = 0.7 Hz, 1H, H-8′), 7.16 (dd, *J* = 8.1 Hz, 0.7 Hz, 1H, H-6′), 6.10 (s, 1H, H-3′), 5.85 (s, 2H, H-11), 5.44 (s, 2H, H-14), 2.40 (s, 3H, CH_3_-7′); ^13^C-NMR (150 MHz, DMSO-*d_6_*) 191.6, 181.3,164.6 161.8, 161.4, 161.4, 152.9, 145.6, 143.7, 141.6, 137.5, 133.9, 133.3, 126.1, 125.3, 124.6, 122.9, 122.7, 119.5, 118.2, 116.5, 116.1, 115.8, 112.6, 90.5, 62.7, 52.1, 21.2; HRMS m/z calcd for C_28_H_20_N_3_O_7_ [M + H]+: 510.1301; found: 510.1301.

*1,8-dihydroxy-3-((4-(((7-methoxy-2-oxo-2H-chromen-4-yl)oxy)methyl)-1H-1,2,3-triazol-1-yl)methyl)anthracene-9,10-dione* (**5g**) yellow solid. Yield: 48%; mp: 281.1–283.3 °C; ^1^H-NMR (500 MHz, DMSO-*d_6_*) *δ* 11.92 (s, 1H, OH-8), 11.91 (s, 1H, OH-1), 8.56 (s, 1H, H-12), 7.82 (dd, *J* = 8.4 Hz, 7.5 Hz, 1H, H-6), 7.72 (dd, *J* = 7.5 Hz, 1.0 Hz, 1H, H-5), 7.64 (d, *J* = 8.9 Hz, 1H, H-5′), 7.60 (d, *J* = 1.5 Hz, 1H, H-4), 7.40 (dd, *J* = 8.4 Hz, 1.0 Hz, 1H, H-7), 7.29 (d, *J* = 1.5 Hz, 1H, H-2), 6.99 (d, *J* = 2.4 Hz, 1H, H-8′), 6.91 (dd, *J* = 8.9 Hz, 2.4 Hz, 1H, H-6′), 6.00 (s, 1H, H-3′), 5.84 (s, 2H, H-11), 5.43 (s, 2H, H-14), 3.84 (s, 3H, OCH_3_-7′); ^13^C-NMR (150 MHz, DMSO-*d_6_*) *δ* 191.6, 181.3, 164.8, 163.0, 162.0, 161.4, 161.3, 154.7, 145.6, 141.6, 137.5, 133.9, 133.3, 126.0, 124.6, 124.0, 122.9, 119.4, 118.2, 116.0, 115.8, 112.3, 108.2, 100.6, 88.7, 62.6, 56.0, 52.1; HRMS *m*/*z* calcd for C_28_H_20_N_3_O_8_ [M + H]^+^: 526.1250; found: 526.1248.

*1,8-dihydroxy-3-((4-(((2-oxo-2H-chromen-6-yl)oxy)methyl)-1H-1,2,3-triazol-1-yl)methyl)anthracene-9,10-dione* (**5h**) yellow solid. Yield: 80%; mp: 248.3–250.2 °C; ^1^H-NMR (500 MHz, DMSO-*d_6_*) *δ* 11.92–11.89 (m, 2H, OH-1, OH-8), 8.43 (s, 1H, H-12), 7.99 (d, *J* = 9.6 Hz, 1H, H-4′), 7.82 (dd, *J* = 8.4 Hz, 7.5 Hz, 1H, H-6), 7.71 (dd, *J* = 7.5 Hz, 1.0 Hz, 1H, H-5), 7.58 (d, *J* = 1.5 Hz, 1H, H-4), 7.42 (d, *J* = 3.0 Hz, 1H, H-5′), 7.40 (dd, *J* = 8.4 Hz, 1.0 Hz, 1H, H-7), 7.35 (d, *J* = 9.1 Hz, 1H, H-8′), 7.28 (dd, *J* = 9.1 Hz, 3.0 Hz, 1H, H-7′), 7.24 (d, *J* = 1.5 Hz, 1H, H-2), 6.49 (d, *J* = 9.6 Hz, 1H, H-3′), 5.81 (s, 2H, H-11), 5.24 (s, 2H, H-14); ^13^C-NMR (125 MHz, DMSO-*d_6_*) *δ* 191.5, 181.2, 161.4, 161.3, 160.1, 154.2, 148.1, 145.6, 144.0, 142.9, 137.5, 133.8, 133.3, 125.4, 124.5, 122.8, 120.1, 119.4, 119.2, 118.2, 117.4, 116.7, 116.0, 115.7, 112.1, 61.7, 52.0; HRMS *m*/*z* calcd for C_27_H_18_N_3_O_7_ [M + H]^+^: 496.1145; found: 496.1141.

*1,8-dihydroxy-3-((4-(((4-methyl-2-oxo-2H-chromen-6-yl)oxy)methyl)-1H-1,2,3-triazol-1-yl)methyl)anthracene-9,10-dione* (**5i**) yellow solid. Yield: 79%; mp: 249.7–251.5 °C; ^1^H-NMR (600 MHz, DMSO-*d_6_*) *δ* 11.91–11.89 (m, 2H, OH-1, OH-8), 8.44 (s, 1H, H-12), 7.81 (dd, *J* = 8.4 Hz, 7.5 Hz, 1H, H-6), 7.70 (dd, *J* = 7.5 Hz, 1.0 Hz, 1H, H-5), 7.56 (d, *J* = 1.6 Hz, 1H, H-4), 7.39 (dd, *J* = 8.4 Hz, 1.0 Hz, 1H, H-7), 7.36 (d, *J* = 2.8 Hz, 1H, H-5′), 7.34 (d, *J* = 9.0 Hz, 1H, H-8′), 7.30 (dd, *J* = 9.0 Hz, 2.8 Hz, 1H, H-7′), 7.24 (d, *J* = 1.6 Hz, 1H, H-2), 6.38 (d, *J* = 1.2 Hz, 1H, H-3′), 5.81 (s, 2H, H-11), 5.29 (s, 2H, H-14), 2.41 (d, *J* = 1.2 Hz, 3H, CH_3_-4′); ^13^C-NMR (150 MHz, DMSO-*d_6_*) 191.6, 181.3, 161.4, 161.4, 159.9, 154.2, 153.1, 147.5, 145.7, 143.0, 137.5, 133.8, 133.3, 125.5, 124.6, 122.8, 120.2, 119.8, 119.5, 118.2, 117.6, 116.0, 115.8, 114.8, 109.6, 61.7, 52.1, 18.3; HRMS *m*/*z* calcd for C_28_H_20_N_3_O_7_ [M + H]+: 510.1301; found: 510.1305.

*1,8-dihydroxy-3-((4-(((2-oxo-2H-chromen-7-yl)oxy)methyl)-1H-1,2,3-triazol-1-yl)methyl)anthracene-9,10-dione* (**5j**) yellow solid. Yield: 94%; mp: 265.2–266.9 °C; ^1^H-NMR (500 MHz, DMSO-*d_6_*) *δ* 11.91–11.88 (m, 2H, OH-1, OH-8), 8.46 (s, 1H, H-12), 7.98 (d, *J* = 9.5 Hz, 1H, H-4′), 7.81 (dd, *J* = 8.4 Hz, 7.5 Hz, 1H, H-6), 7.70 (dd, *J* = 7.5 Hz, 0.9 Hz, 1H, H-5), 7.63 (d, *J* = 8.7 Hz, 1H, H-5′), 7.58 (d, *J* = 1.4 Hz, 1H, H-4), 7.39 (dd, *J* = 8.4 Hz, 0.9 Hz, 1H, H-7), 7.25 (d, *J* = 1.4 Hz, 1H, H-2), 7.15 (d, *J* = 2.4 Hz, 1H, H-8′), 7.02 (dd, *J* = 8.7 Hz, 2.4 Hz, 1H, H-6′), 6.29 (d, *J* = 9.5 Hz, 1H, H-3′), 5.81 (s, 2H, H-11), 5.31 (s, 2H, H-14); ^13^C-NMR (125 MHz, DMSO-*d_6_*) *δ* 191.5, 181.2, 161.4, 161.3, 161.0, 160.2, 155.3, 145.6, 144.3, 142.5, 137.5, 133.8, 133.2, 129.5, 125.6, 124.5, 122.8, 119.4, 118.2, 116.0, 115.7, 112.9, 112.7, 112.6, 101.6, 61.6, 52.1; HRMS *m*/*z* calcd for C_27_H_18_N_3_O_7_ [M + H]^+^: 496.1145; found: 496.1146.

*1,8-dihydroxy-3-((4-(((4-methyl-2-oxo-2H-chromen-7-yl)oxy)methyl)-1H-1,2,3-triazol-1-yl)methyl)anthracene-9,10-dione* (**5k**) yellow solid. Yield: 88%; mp: 233.7–235.6 °C; ^1^H-NMR (600 MHz, DMSO-*d_6_*) *δ* 11.92–11.89 (m, 2H, OH-1, OH-8), 8.47 (s, 1H, H-12), 7.81 (dd, *J* = 8.4 Hz, 7.5 Hz, 1H, H-6), 7.70 (dd, *J* =7.5 Hz, 0.9 Hz, 1H, H-5), 7.68 (d, *J* = 8.8 Hz, 1H, H-5′), 7.58 (d, *J* = 1.3 Hz, 1H, H-4), 7.39 (dd, *J* = 8.4 Hz, 0.9 Hz, 1H, H-7), 7.26 (d, *J* = 1.3 Hz, 1H, H-2), 7.15 (d, *J* = 2.5 Hz, 1H, H-8′), 7.03 (dd, *J* = 8.8 Hz, 2.5 Hz, 1H, H-6′), 6.21 (d, *J* = 1.0 Hz, 1H, H-3′), 5.81 (s, 2H, H-11), 5.31 (s, 2H, H-14), 2.39 (d, *J* = 1.0 Hz, 3H, CH_3_-4′); ^13^C-NMR (150 MHz, DMSO-*d_6_*) *δ* 191.6, 181.3, 161.4, 161.4, 161.0, 160.2, 154.7, 153.5, 145.6, 142.5, 137.5, 133.9, 133.3, 126.6, 125.7, 124.6, 122.9, 119.5, 118.2, 116.0, 115.8, 113.4, 112.7, 111.4, 101.6, 61.6, 52.1, 18.2; HRMS *m*/*z* calcd for C_28_H_20_N_3_O_7_ [M + H]^+^: 510.1301; found: 510.1303.

*1,8-dihydroxy-3-((4-(((3-cyano-2-oxo-2H-chromen-7-yl)oxy)methyl)-1H-1,2,3-triazol-1-yl)methyl)anthracene-9,10-dione* (**5l**) yellow solid. Yield: 54%; mp: 258.4–260.2 °C; ^1^H-NMR (600 MHz, DMSO-*d_6_*) *δ* 11.91–11.88 (m, 2H, OH-1, OH-8), 8.85 (s, 1H, H-4′), 8.49 (s, 1H, H-12), 7.80 (dd, *J* = 8.4 Hz, 7.4 Hz, 1H, H-6), 7.73 (d, *J* = 8.8 Hz, 1H, H-5′), 7.69 (d, *J* = 7.4 Hz, 1H, H-5), 7.55 (d, *J* = 1.3 Hz, 1H, H-4), 7.39 (d, *J* = 8.4 Hz, 1H, H-7), 7.29 (d, *J* = 2.3 Hz, 1H, H-8′), 7.25 (d, *J* = 1.3 Hz, 1H, H-2), 7.13 (dd, *J* = 8.8 Hz, 2.3 Hz, 1H, H-6′), 5.82 (s, 2H, H-11), 5.38 (s, 2H, H-14); ^13^C-NMR (150 MHz, DMSO-*d_6_*) *δ* 191.5, 181.2, 163.9, 161.4, 161.4, 157.4, 156.5, 153.2, 145.6, 142.1, 137.5, 133.8, 133.2, 131.4, 125.9, 124.6, 122.9, 119.4, 118.1, 116.0, 115.7, 115.1, 114.5, 111.6, 101.9, 97.8, 62.0, 52.1; HRMS *m*/*z* calcd for C_28_H_17_N_4_O_7_ [M + H]^+^: 521.1097; found: 521.1090.

*1,8-dihydroxy-3-((4-(2-((2-oxo-2H-chromen-4-yl)oxy)ethyl)-1H-1,2,3-triazol-1-yl)methyl)anthracene-9,10-dione* (**5m**) yellow solid. Yield: 82%; mp: 250.4–251.8 °C; ^1^H-NMR (600 MHz, DMSO-*d_6_*) *δ* 11.90 (s, 1H, OH-8), 11.88 (s, 1H, OH-1), 8.22 (s, 1H, H-12), 7.81 (dd, *J* = 8.4 Hz, 7.7 Hz, 1H, H-6), 7.69–7.66 (m, 2H, H-5, H-5′), 7.56 (ddd, *J* = 8.4 Hz, 7.4 Hz, 1.6 Hz, 1H, H-7′), 7.48 (d, *J* = 1.6 Hz, 1H, H-4), 7.39 (dd, *J* = 8.4 Hz, 0.9 Hz, 1H, H-7), 7.31 (dd, *J* = 8.4 Hz, 0.9 Hz, 1H, H-8′), 7.25 (ddd, *J* = 7.8 Hz, 7.4 Hz, 0.9 Hz, 1H, H-6′), 7.21 (d, *J* = 1.6 Hz, 1H, H-2), 5.94 (s, 1H, H-3′), 5.76 (s, 2H, H-11), 4.47 (t, *J* = 6.2 Hz, 2H, H-15), 3.25 (t, *J* = 6.2 Hz, 2H, H-14); ^13^C-NMR (150 MHz, DMSO-*d_6_*) 191.5, 181.2, 164.8, 161.7, 161.4, 161.3, 152.7, 146.0, 143.8, 137.5, 133.7, 133.2, 132.6, 124.6, 124.1, 123.8, 122.8, 122.7, 119.4, 118.0, 116.4, 116.0, 115.6, 115.2, 90.8, 68.4, 51.9, 24.9; HRMS *m*/*z* calcd for C_28_H_20_N_3_O_7_ [M + H]^+^: 510.1301; found: 510.1298.

#### 3.2.5. Procedure for the Synthesis of Compound **6a** and **6b**

Iodomethane (2.0 mmol) or benzyl bromide (1.0 mmol) and anhydrous K_2_CO_3_ (2.0 mmol) were added to a solution of **4** (0.2 mmol) in DMF (3 mL). The reaction was heated to 60 °C for 4 h. Upon completion, the reaction was cooled down to room temperature, and then the reaction mixture was diluted with water and extracted three times with CH_2_Cl_2_. The organic layers were dried with dry Na_2_SO_4_, they were filtered, and the filtrate was removed under reduced pressure. The residue was purified by column chromatography to give compound **6a** or **6b**.

*3-(azidomethyl)-1,8-dimethoxyanthracene-9,10-dione* (**6a**) yellow solid. Yield: 87%; mp: 133.6–135.0 °C; ^1^H-NMR (600 MHz, CDCl_3_) *δ* 7.83 (dd, *J* = 7.7 Hz, 0.8 Hz, 1H, H-5), 7.75 (d, *J* = 1.3 Hz, 1H, H-4), 7.64 (dd, *J* = 8.1 Hz, 7.7 Hz, 1H, H-6), 7.31 (d, *J* = 8.1 Hz, 1H, H-7), 7.25 (d, *J* = 1.3 Hz, 1H, H-2), 4.50 (s, 2H, H-11), 4.03 (s, 3H, OCH_3_-8), 4.01 (s, 3H, OCH_3_-1); ^13^C-NMR (150 MHz, CDCl_3_) 183.9, 182.6, 160.2, 159.7, 142.0, 135.2, 134.7, 134.2, 124.0, 123.7, 119.1, 118.3, 118.0, 116.9, 56.8, 56.7, 54.3.

*3-(azidomethyl)-1,8-bis(benzyloxy)anthracene-9,10-dione* (**6b**) yellow solid. Yield: 67%; mp: 133.9–135.5 °C; ^1^H-NMR (600 MHz, CDCl_3_) *δ* 7.85 (dd, *J* = 7.7 Hz, 0.7 Hz, 1H, H-5), 7.77 (d, *J* = 0.9 Hz, 1H, H-4), 7.66–7.62 (m, 4H, CH_2_C_6_H_5_-1, CH_2_C_6_H_5_-8), 7.60 (dd, *J* = 8.1 Hz, 7.7 Hz, 1H, H-6), 7.42–7.38 (m, 4H, CH_2_C_6_H_5_-1, CH_2_C_6_H_5_-8), 7.36–7.33 (m, 3H, H-7, CH_2_C_6_H_5_-1, CH_2_C_6_H_5_-8), 7.30 (d, *J* = 0.9 Hz, 1H, H-2), 5.34 (s, 2H, CH_2_C_6_H_5_-8), 5.33 (s, 2H, CH_2_C_6_H_5_-1), 4.46 (s, 2H, H-11); ^13^C-NMR (150 MHz, CDCl_3_) 183.8, 181.9, 158.9, 158.4, 141.9, 136.6, 136.4, 135.3, 134.9, 134.0, 128.8, 128.7, 128.0, 127.9, 126.9, 126.9, 124.8, 124.5, 120.4, 119.6, 118.9, 118.5, 71.3, 71.2, 54.2.

#### 3.2.6. Procedure for the Synthesis of Compound **6c** and **6d**

Acetyl chloride (0.6 mmol) or benzoyl chloride (0.6 mmol), 4-dimethylaminopyridine (0.04 mmol) and *N*,*N*-diisopropylethylamine (0.6 mmol) were added to a solution of **4** (0.2 mmol) in CH_2_Cl_2_ (3 mL). After being stirred at room temperature for 4 h, the reaction mixture was diluted with water and extracted three times with CH_2_Cl_2_. The organic layers were dried with anhydrous Na_2_SO_4_, and the solvent was removed under reduced pressure. The residue was purified by silica gel column chromatography to give compound **6c** or **6d**.

*3-(azidomethyl)-9,10-dioxo-9,10-dihydroanthracene-1,8-diyl diacetate* (**6c**) yellow solid. Yield: 89%; mp: 161.2–162.8 °C; ^1^H-NMR (600 MHz, CDCl_3_) *δ* 8.23 (dd, *J* = 7.8 Hz, 1.3 Hz, 1H, H-5), 8.15 (d, *J* = 1.8 Hz, 1H, H-4), 7.78 (dd, *J* = 8.0 Hz, 7.8 Hz, 1H, H-6), 7.42 (dd, *J* = 8.0 Hz, 1.3 Hz, 1H, H-7), 7.39 (d, *J* = 1.8 Hz, 1H, H-2), 4.56 (s, 2H, H-11), 2.46 (s, 3H, COCH_3_-8), 2.45 (s, 3H, COCH_3_-1); ^13^C-NMR (150 MHz, CDCl_3_) 181.8, 180.5, 169.6, 169.5, 150.7, 150.2, 143.4, 134.9, 134.8, 134.5, 130.6, 129.0, 125.7, 125.6, 125.1, 124.3, 53.6, 21.3, 21.2.

*3-(azidomethyl)-9,10-dioxo-9,10-dihydroanthracene-1,8-diyl dibenzoate* (**6d**) yellow solid. Yield: 83%; mp: 175.6–176.8 °C; ^1^H-NMR (600 MHz, CDCl_3_) *δ* 8.26 (dd, *J* = 7.8 Hz, 1.1 Hz, 1H, H-5), 8.18 (d, *J* = 1.7 Hz, 1H, H-4), 8.03–8.00 (m, 4H, COC_6_H_5_-1, COC_6_H_5_-8), 7.81 (dd, *J* = 8.2 Hz, 7.8 Hz, 1H, H-6), 7.55 (dd, *J* = 8.2 Hz, 1.1 Hz, 1H, H-7), 7.53–7.48 (m, 3H, H-2, COC_6_H_5_-1, COC_6_H_5_-8), 7.29–7.25 (m, 4H, COC_6_H_5_-1, COC_6_H_5_-8), 4.58 (s, 2H, H-11); ^13^C-NMR (150 MHz, CDCl_3_) 182.0, 180.6, 165.5, 165.4, 150.7, 150.3, 143.2, 134.8, 134.7, 134.5, 133.4, 133.3, 130.6, 130.3, 130.3, 129.4, 129.3, 129.1, 128.4, 128.3, 126.1, 125.6, 125.6, 124.2, 53.7.

#### 3.2.7. Procedure for the Synthesis of Compound **6e**

*p*-Toluenesulfonyl chloride (82 mg, 0.43 mmol) and anhydrous K_2_CO_3_ (70 mg, 0.51 mmol) were added to a solution of **4** (50 mg, 0.17 mmol) in acetone (5 mL). The reaction was heated to 60 °C for 5 h. Upon completion, the reaction was cooled down to room temperature, and then the reaction mixture was diluted with water and extracted three times with CH_2_Cl_2_. The organic layers were dried with dry Na_2_SO_4_, they were filtered, and the filtrate was removed under reduced pressure. The residue was purified by column chromatography (petroleum ether:ethyl acetate = 5:1) to give compound **6e**. 

*3-(azidomethyl)-9,10-dioxo-9,10-dihydroanthracene-1,8-diyl bis(4-methylbenzenesulfonate)* (**6e**) yellow solid. Yield: 83%; mp: 131.3–133.1 °C; ^1^H-NMR (600 MHz, CDCl_3_) *δ* 8.19 (dd, *J* = 7.7 Hz, 1.3 Hz, 1H, H-5), 8.12 (d, *J* = 1.7 Hz, 1H, H-4), 7.95 (d, *J* = 8.3 Hz, 2H, SO_2_C_6_H_4_-8), 7.92 (d, *J* = 8.3 Hz, 2H, SO_2_C_6_H_4_-1), 7.72 (dd, *J* = 8.1 Hz, 7.7 Hz, 1H, H-6), 7.65 (dd, *J* = 8.1 Hz, 1.3 Hz, 1H, H-7), 7.62 (d, *J* = 1.7 Hz, 1H, H-2), 7.36–7.31 (m, 4H, SO_2_C_6_H_4_-1, SO_2_C_6_H_4_-8), 4.52 (s, 2H, H-11), 2.43 (s, 3H, C_6_H_4_CH_3_-8), 2.43 (s, 3H, C_6_H_4_CH_3_-1); ^13^C NMR (150 MHz, CDCl_3_) 181.4, 178.6, 147.8, 147.4, 146.1, 145.9, 143.0, 134.9, 134.5, 134.4, 132.3, 132.2, 130.3, 130.1, 130.0, 129.2, 129.1, 128.9, 127.7, 127.2, 126.2, 124.9, 53.5, 21.9, 21.9. 

#### 3.2.8. General Procedures for the Synthesis of Aloe-Emodin–Coumarin Hybrids (**7a**–**e**)

Compound **2d** (0.18 mmol) and copper(I) thiophene-2-carboxylate (0.03 mmol) were added to a solution of compound **6a** (0.15 mmol) in CH_2_Cl_2_ (5 mL). After being stirred at room temperature for 6 h, the reaction mixture was concentrated, and the residue was purified by silica gel column chromatography to give compounds **7a**–**e**.

*3-((4-(((6-chloro-2-oxo-2H-chromen-4-yl)oxy)methyl)-1H-1,2,3-triazol-1-yl)methyl)-1,8-dimethoxyanthracene-9,10-dione* (**7a**) yellow solid. Yield: 82%; mp: 251.8–252.6 °C; ^1^H-NMR (600 MHz, DMSO-*d_6_*) *δ* 8.59 (s, 1H, H-12), 7.73 (dd, *J* = 8.3 Hz, 7.5 Hz, 1H, H-6), 7.69 (dd, *J* = 8.8 Hz, 2.6 Hz, 1H, H-7′), 7.66–7.63 (m, 2H, H-5, H-5′), 7.57 (d, *J* = 0.7 Hz, 1H, H-4), 7.52 (d, *J* = 8.3 Hz, 1H, H-7), 7.49 (d, *J* = 0.7 Hz, 1H, H-2), 7.45 (d, *J* = 8.8 Hz, 1H, H-8′), 6.24 (s, 1H, H-3′), 5.83 (s, 2H, H-11), 5.46 (s, 2H, H-14), 3.91 (s, 3H, OCH_3_-8), 3.90 (s, 3H, OCH_3_-1); ^13^C-NMR (150 MHz, DMSO-*d_6_*) 183.0, 180.9, 163.2, 161.1, 159.0, 158.8, 151.5, 142.3, 141.3, 134.5, 134.4, 134.0, 132.6, 128.3, 126.0, 123.3, 123.1, 122.0, 119.1, 118.8, 118.2, 118.1, 117.0, 116.6, 92.3, 63.1, 56.5, 56.4, 52.5; HRMS *m*/*z* calcd for C_29_H_21_ClN_3_O_7_ [M + H]^+^: 558.1068; found: 558.1070.

*1,8-bis(benzyloxy)-3-((4-(((6-chloro-2-oxo-2H-chromen-4-yl)oxy)methyl)-1H-1,2,3-triazol-1-yl)methyl)anthracene-9,10-dione* (**7b**) yellow solid. Yield: 40%; mp: 258.3–259.8 °C; ^1^H-NMR (600 MHz, DMSO-*d_6_*) *δ* 8.57 (s, 1H, H-12), 7.76 (dd, *J* = 8.3 Hz, 7.5 Hz, 1H, H-6), 7.72–7.66 (m, 4H, H-5, H-5′, H-4, H-7′), 7.65–7.60 (m, 5H, H-7, CH_2_C_6_H_5_-1, CH_2_C_6_H_5_-8), 7.56 (d, *J* = 1.0 Hz, 1H, H-2), 7.46 (d, *J* = 8.7 Hz, 1H, H-8′), 7.43–7.38 (m, 4H, CH_2_C_6_H_5_-1, CH_2_C_6_H_5_-8), 7.37–7.33 (m, 2H, CH_2_C_6_H_5_-1, CH_2_C_6_H_5_-8), 6.26 (s, 1H, H-3′), 5.83 (s, 2H, H-11), 5.46 (s, 2H, H-14), 5.33 (s, 2H, CH_2_C_6_H_5_-8), 5.32 (s, 2H, CH_2_C_6_H_5_-1); ^13^C-NMR (150 MHz, DMSO-*d_6_*) 182.9, 180.9, 163.2, 161.1, 158.0, 157.7, 151.5, 142.2, 141.3, 136.9, 136.6, 134.6, 134.4, 134.2, 132.6, 128.4, 128.3, 127.8, 127.7, 127.0, 126.9, 126.0, 123.8, 123.6, 122.0, 120.6, 119.7, 118.7, 118.7, 117.5, 116.6, 92.3, 70.3, 70.1, 63.1, 52.4; HRMS *m*/*z* calcd for C_41_H_29_ClN_3_O_7_ [M + H]^+^: 710.1694; found: 710.1690.

*3-((4-(((6-chloro-2-oxo-2H-chromen-4-yl)oxy)methyl)-1H-1,2,3-triazol-1-yl)methyl)-9,10-dioxo-9,10-dihydroanthracene-1,8-diyl diacetate* (**7c**) yellow solid. Yield: 55%; mp: 245.6–247.2 °C; ^1^H-NMR (600 MHz, DMSO-d_6_) δ 8.61 (s, 1H, H-12), 8.11 (d, *J* = 7.6 Hz, 1H, H-5), 7.98 (s, 1H, H-4), 7.93 (dd, *J* = 7.9 Hz, 7.6 Hz, 1H, H-6), 7.71 (dd, *J* = 8.7 Hz, 2.2 Hz, 1H, H-7′), 7.69 (d, *J* = 2.2 Hz, 1H, H-5′), 7.63 (d, *J* =7.9 Hz, 1H, H-7), 7.53 (s, 1H, H-2), 7.48 (d, *J* = 8.7 Hz, 1H, H-8′), 6.25 (s, 1H, H-3′), 5.92 (s, 2H, H-11), 5.47 (s, 2H, H-14), 2.40–2.35 (m, 6H, COCH_3_-1, COCH_3_-8); ^13^C-NMR (150 MHz, DMSO-d_6_) 181.1, 180.3, 169.1, 169.1, 163.2, 161.1, 151.5, 149.9, 149.6, 143.6, 141.4, 135.4, 134.5, 134.1, 132.6, 130.7, 129.2, 128.3, 126.2, 125.2, 125.0, 124.8, 123.7, 122.0, 118.8, 116.6, 92.3, 63.1, 51.8, 20.9, 20.9; HRMS *m*/*z* calcd for C_31_H_21_ClN_3_O_9_ [M + H]^+^: 614.0966; found: 614.0961.

*3-((4-(((6-chloro-2-oxo-2H-chromen-4-yl)oxy)methyl)-1H-1,2,3-triazol-1-yl)methyl)-9,10-dioxo-9,10-dihydroanthracene-1,8-diyl dibenzoate* (**7d**) yellow solid. Yield: 83%; mp: 231.1–232.6 °C; ^1^H-NMR (600 MHz, DMSO-d_6_) δ 8.63 (s, 1H, H-12), 8.16 (dd, *J* = 7.8 Hz, 1.1 Hz, 1H, H-5), 8.04 (d, *J* = 1.6 Hz, 1H, H-4), 7.98 (dd, *J* = 8.0 Hz, 7.8 Hz, 1H, H-6), 7.94–7.91 (m, 4H, COC_6_H_5_-1, COC_6_H_5_-8), 7.78 (dd, *J* = 8.0 Hz, 1.1 Hz, 1H, H-7), 7.73 (d, *J* = 1.6 Hz, 1H, H-2), 7.70 (dd, *J* = 8.8 Hz, 2.6 Hz, 1H, H-7′), 7.68 (d, *J* = 2.6 Hz, 1H, H-5′), 7.65–7.61 (m, 2H, COC_6_H_5_-1, COC_6_H_5_-8), 7.46 (d, *J* = 8.8 Hz, 1H, H-8′), 7.43–7.37 (m, 4H, COC_6_H_5_-1, COC_6_H_5_-8), 6.25 (s, 1H, H-3′), 5.95 (s, 2H, H-11), 5.47 (s, 2H, H-14); ^13^C-NMR (150 MHz, DMSO-d_6_) 181.2, 180.6, 164.6, 164.5, 163.2, 161.1, 151.5, 149.5, 149.2, 143.4, 141.4, 135.3, 134.6, 134.2, 133.8, 133.7, 132.6, 130.7, 129.7, 129.7, 129.2, 128.8, 128.7, 128.7, 128.6, 128.3, 126.2, 125.8, 125.4, 125.0, 123.8, 122.0, 118.7, 116.6, 92.3, 63.1, 51.9; HRMS *m*/*z* calcd for C_41_H_25_ClN_3_O_9_ [M + H]^+^: 738.1279; found: 738.1281.

*3-((4-(((6-chloro-2-oxo-2H-chromen-4-yl)oxy)methyl)-1H-1,2,3-triazol-1-yl)methyl)-9,10-dioxo-9,10-dihydroanthracene-1,8-diyl bis(4-methylbenzenesulfonate)* (**7e**) yellow solid. Yield: 95%; mp: 195.6–197.1 °C; ^1^H-NMR (600 MHz, DMSO-d_6_) δ 8.56 (s, 1H, H-12), 8.10 (dd, *J* = 7.8 Hz, 1.1 Hz, 1H, H-5), 8.03 (d, *J* = 1.6 Hz, 1H, H-4), 7.89 (dd, *J* = 8.1 Hz, 7.8 Hz, 1H, H-6), 7.80 (d, *J* = 8.3 Hz, 2H, SO_2_C_6_H_4_-8), 7.75 (d, *J* = 8.3 Hz, 2H, SO_2_C_6_H_4_-1), 7.69–7.65 (m, 2H, H-5′, H-7′), 7.57 (dd, *J* = 8.1 Hz, 1.1 Hz, 1H, H-7), 7.49 (d, *J* = 1.6 Hz, 1H, H-2), 7.45 (d, *J* = 8.7 Hz, 1H, H-8′), 7.41 (d, *J* = 8.1 Hz, 2H, SO_2_C_6_H_4_-8), 7.39 (d, *J* = 8.1 Hz, 2H, SO_2_C_6_H_4_-1), 6.28 (s, 1H, H-3′), 5.92 (s, 2H, H-11), 5.49 (s, 2H, H-14), 2.37 (s, 3H, C_6_H_4_CH_3_-8), 2.36 (s, 3H, C_6_H_4_CH_3_-1); ^13^C-NMR (150 MHz, DMSO-d_6_) 180.5, 178.2, 163.2, 161.1, 151.5 146.7, 146.3, 146.1, 146.0, 143.3, 141.4, 135.1, 134.8, 134.5, 132.6, 131.6, 131.4, 130.2, 130.2, 129.5, 128.5, 128.4, 128.3, 127.7, 127.2, 126.5, 126.1, 125.8, 124.5, 122.0, 118.7, 116.6, 92.3, 63.0, 51.7, 21.2, 21.2; HRMS *m*/*z* calcd for C_41_H_29_ClN_3_O_11_S_2_ [M + H]^+^: 838.0932; found: 838.0931.

### 3.3. Evaluation of the Biological Activity

A549, HepG2, MCF-7 and HCT-8 were obtained from the Cell Resource Center, Peking Union Medical College (which is the headquarters of the National Infrastructure of Cell Line Resource, NSTI) (Beijing, China), SGC-7901 was purchased from Beyotime (Beijing, China), and Hk-2 was obtained from the American Type Culture Collection (ATCC) (Manassas, VA, USA). The antiproliferative activity of the synthetic compounds was evaluated by the MTT assay in vitro, with etoposide as a positive control. In brief, the cells were seeded into 96-well plates at 5 × 10^3^ cells/well and were cultured overnight for adherence. The test compounds were prepared from 10 mM or 5 mM stock solution in DMSO according to preliminary screening results. Then, these stocks solution were serially diluted with DMEM medium with a final DMSO concentration no greater than 0.5%. The concentration was set to 10 μM, 5 μM, 2.5 μM, 1.25 μM, 0.625μM, 0.3125 μM and 0.15625 μM for potent compounds and 40 μM, 20 μM, 10 μM, 5 μM and 2.5 μM for less potent compounds. The cells were treated with different concentrations of test compounds for 48 h. After that, 20 μL MTT (5 mg/mL) was added to each well, and the plates were further incubated for 4 h. The medium was carefully removed, and the formazan crystals were dissolved in 150 μL DMSO. The absorbance at 490 nm was measured on a microplate reader (TECAN Infinite M1000, Austria). The cell viability was determined in terms of IC_50_ value.

## 4. Conclusions

In summary, a new series of aloe-emodin–coumarin hybrids were designed, synthesized and evaluated for their anticancer activity against five human cancer cell lines. Some of the synthesized compounds showed potent antiproliferative activity against one or more cell lines. Particularly, compound **5d** exhibited a broad spectrum of antitumor activity against all tested cancer cell lines. Preliminary SAR analysis showed that the introduction of the chloro group in the 5-position of coumarin for the derivatives linked at the 4-position of coumarin was beneficial for the antitumor activity.

## Figures and Tables

**Table 1:** The structures, yields and melting points of target compounds **5a**–**m** and **7a**–**e**.

Comp.	Structure	Yield	M.p.	Comp.	Structure	Yield	M.p.
**5a**	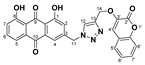	57%	252.3–254.1 °C	**5j**	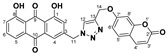	94%	265.2–266.9 °C
**5b**	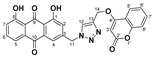	46%	272.5–273.8 °C	**5k**	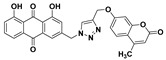	88%	233.7–235.6 °C
**5c**	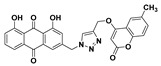	49%	274.9–276.9 °C	**5l**	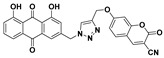	54%	258.4–260.2 °C
**5d**	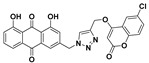	82%	286.3–288.1 °C	**5m**	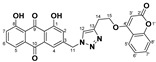	82%	250.4–251.8 °C
**5e**	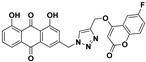	58%	245.9–247.8 °C	**7a**	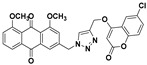	82%	251.8–252.6 °C
**5f**	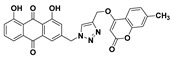	85%	283.3–284.8 °C	**7b**	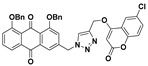	40%	258.3–259.8 °C
**5g**	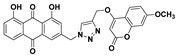	48%	281.1–283.3 °C	**7c**	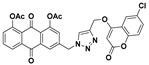	55%	245.6–247.2 °C
**5h**	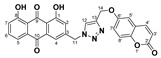	80%	248.3–250.2 °C	**7d**	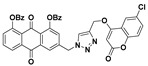	83%	231.1–232.6 °C
**5i**	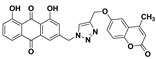	79%	249.7–251.5 °C	**7e**	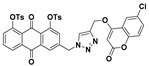	95%	195.6–197.1 °C

**Table 2:** In vitro antitumor activities of target compounds **5a**–**m** and **7a**–**e**.

Compound	IC_50_ (μmol/L) ^a, b^					
	A549	SGC-7901	HepG2	MCF-7	HCT-8	Hk-2
**5a**	2.57 ± 0.48	3.28 ± 0.67	7.06 ± 0.27	2.34 ± 0.48	1.56 ± 0.45	<1.0
**5b**	>40	>40	21.86 ± 0.75	25.27 ± 6.072	>40	-
**5c**	>40	19.30 ± 1.28	14.23 ± 2.38	31.34 ± 0.62	34.07 ± 1.38	-
**5d**	**1.12 ± 0.09**	**0.90 ± 0.12**	**0.99 ± 0.12**	**1.59** **± 0.58**	**0.48 ± 0.06**	<1.0
**5e**	>40	>40	>40	>40	>40	
**5f**	3.82 ± 0.31	2.17 ± 0.24	1.02 ± 0.44	1.68 ± 0.21	4.49 ± 0.24	<10
**5g**	4.05 ± 1.99	9.04 ± 1.17	5.01 ± 1.27	3.60 ± 0.92	5.79 ± 1.24	<10
**5h**	>40	>40	14.95 ± 1.52	>40	>40	-
**5i**	>40	16.92 ± 0.12	6.96 ± 1.02	>40	>40	-
**5j**	15.34 ± 4.87	10.01 ± 1.21	4.68 ± 0.66	11.16 ± 3.75	24.23 ± 1.07	-
**5k**	>40	13.45 ± 0.93	2.04 ± 0.71	9.26 ± 1.00	>40	-
**5l**	>40	>40	>40	21.87 ± 2.16	>40	-
**5m**	>40	10.67 ± 0.40	3.56 ± 1.03	2.35 ± 0.65	2.84 ± 1.88	-
**7a**	>40	13.95 ± 2.79	3.14 ± 1.33	3.12 ± 1.31	>40	-
**7b**	>40	>40	>40	>40	>40	-
**7c**	>40	1.29 ± 0.32	1.94 ± 0.74	1.56 ± 1.00	3.53 ± 0.91	<10
**7d**	>40	32.09 ± 1.23	>40	>40	>40	-
**7e**	>40	>40	>40	>40	>40	-
**aloe-emodin**	16.38 ± 2.82	10.78 ± 3.54	3.61 ± 0.81	10.8 ± 0.41	16.33 ± 0.67	-
**etoposide**	3.18 ± 1.36	8.30 ± 1.09	5.77 ± 0.99	5.45 ± 1.14	3.40 ± 0.42	-

^a^ IC_50_ values are shown as mean ± SD from the average of three replicates; ^b^ IC_50_: concentration that causes a 50% reduction in cell growth; “-”: not detected.

## Data Availability

Not applicable.

## Supplementary Materials

The following supporting information can be downloaded at: https://www.mdpi.com/article/10.3390/molecules27196153/s1, ^1^H-NMR and ^13^C-NMR spectra of the synthesized compounds.

## Sample Availability

Samples of the compounds are not available from the authors.
