# Supplementary material for: The Synthesis and Biological Evaluation of Aloe-Emodin-Coumarin Hybrids as Potential Antitumor Agents
Source: Molecules. 2022 Sep 20;27(19):6153. doi: 10.3390/molecules27196153 (PMC9571363; doi:10.3390/molecules27196153)
Supplement: Supplementary file 1 [file molecules-27-06153-s001.zip › molecules-1877704-supplementary.pdf]

Supplementary Materials

The Synthesis and Biological Evaluation of Aloe-Emodin-Coumarin Hybrids as Potential Antitumor Agents

Hai Shang [1], Yue Hu [1], Jingrong Li [1,2], Lingyu Li [1], Yu Tian [1], Xiaoxue Li [1,3], Qi Wu [1,4] and Zhongmei Zou [1,*]

[1] Institute of Medicinal Plant Development, Chinese Academy of Medical Sciences and Peking Union Medical College, Beijing 100193, China
[2] Basic Medical Sciences, Guizhou Medical University, Guiyang 550025, China
[3] School of Traditional Chinese Materia Medica, Shenyang Pharmaceutical University, Shenyang 110016, China
[4] School of Pharmaceutical Engineering, Shenyang Pharmaceutical University, Shenyang 110016, China
* Correspondence: zmzou@implad.ac.cn; Tel.: +86-10-5783-3290

1. NMR spectrum

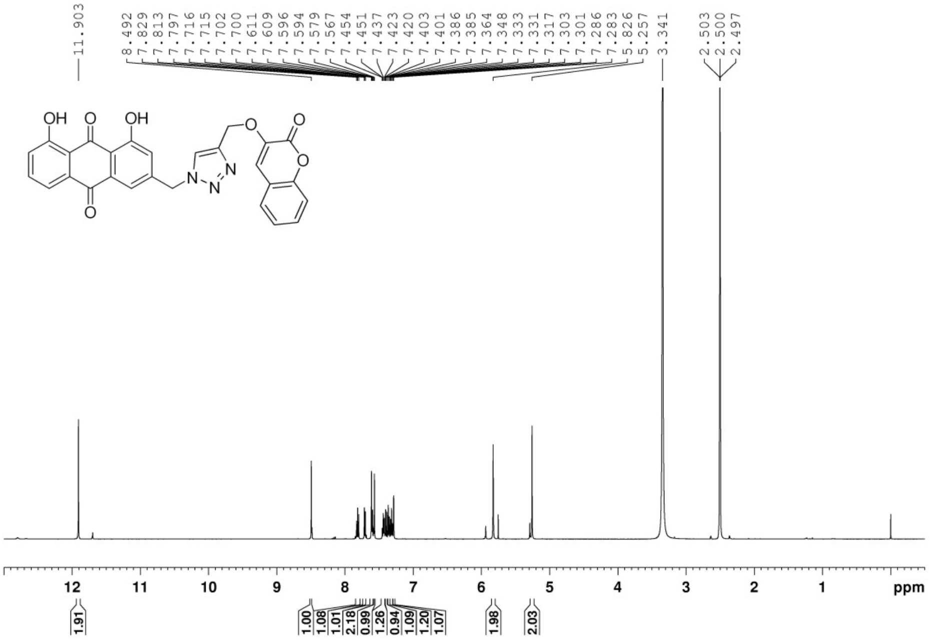

^{1}H-NMR Spectrum for 5a (DMSO-*d*[6], 500 MHz).

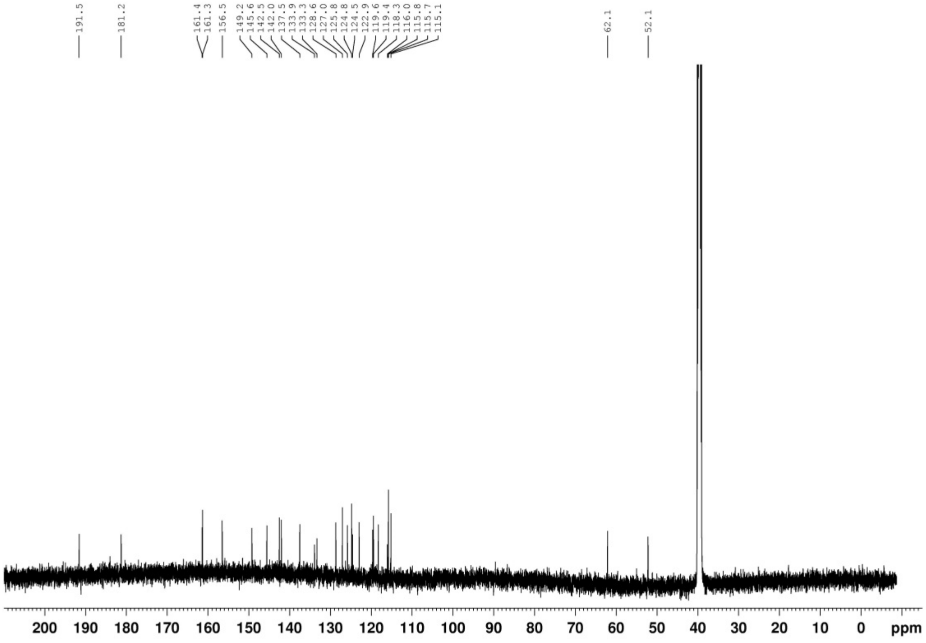

^{13}C-NMR Spectrum for 5a (DMSO-*d*[6], 125 MHz).

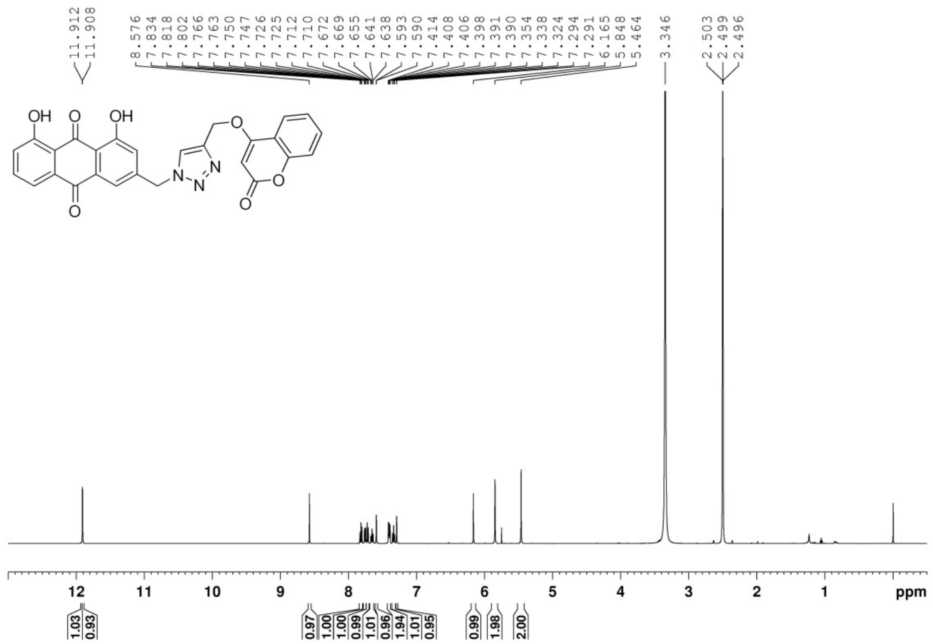

^{1}H-NMR Spectrum for 5b (DMSO-d_6, 500 MHz).

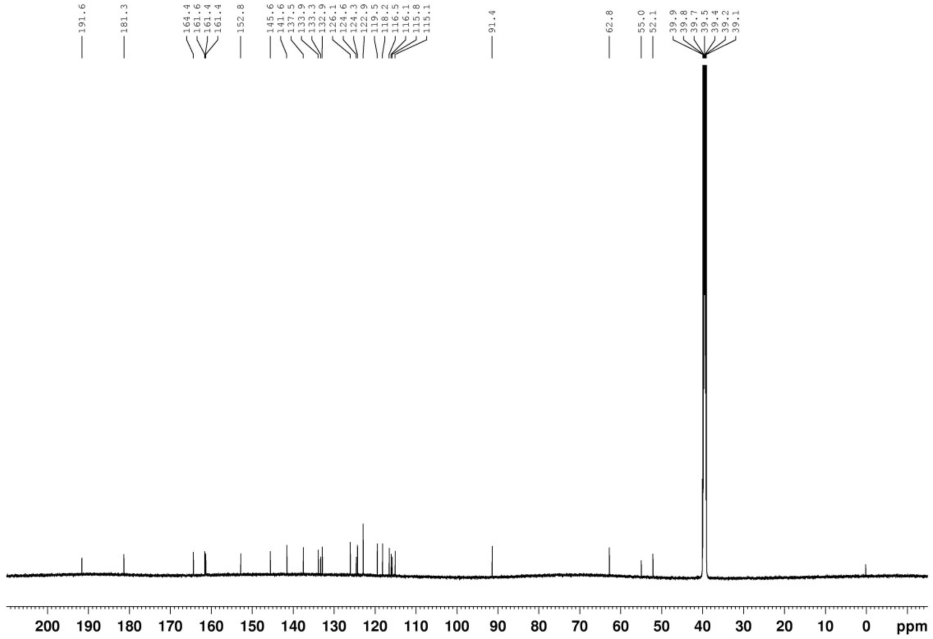

^{13}C-NMR Spectrum for 5b (DMSO-d_6, 150 MHz).

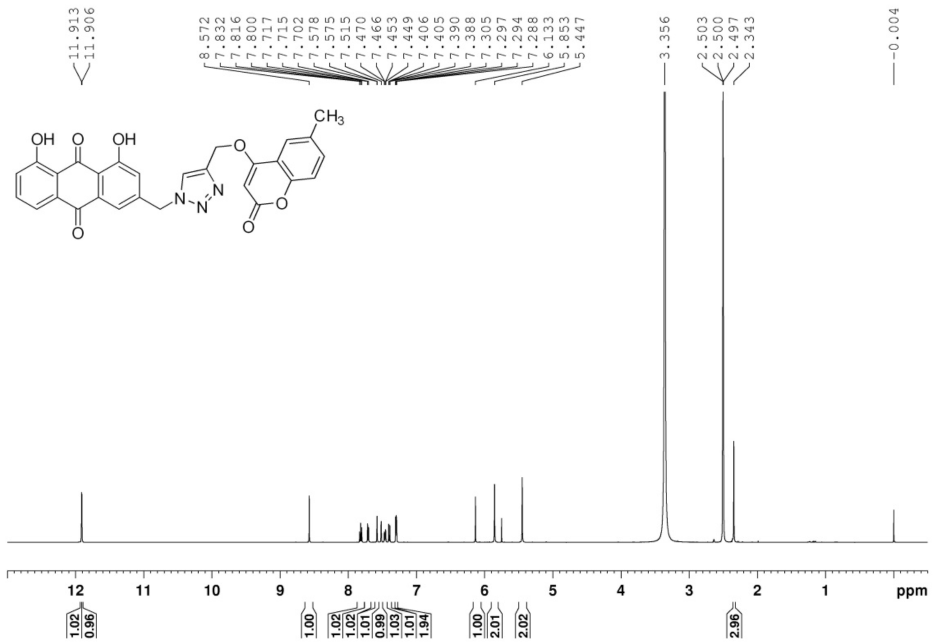

^{1}H-NMR Spectrum for 5c (DMSO-*d*6, 500 MHz).

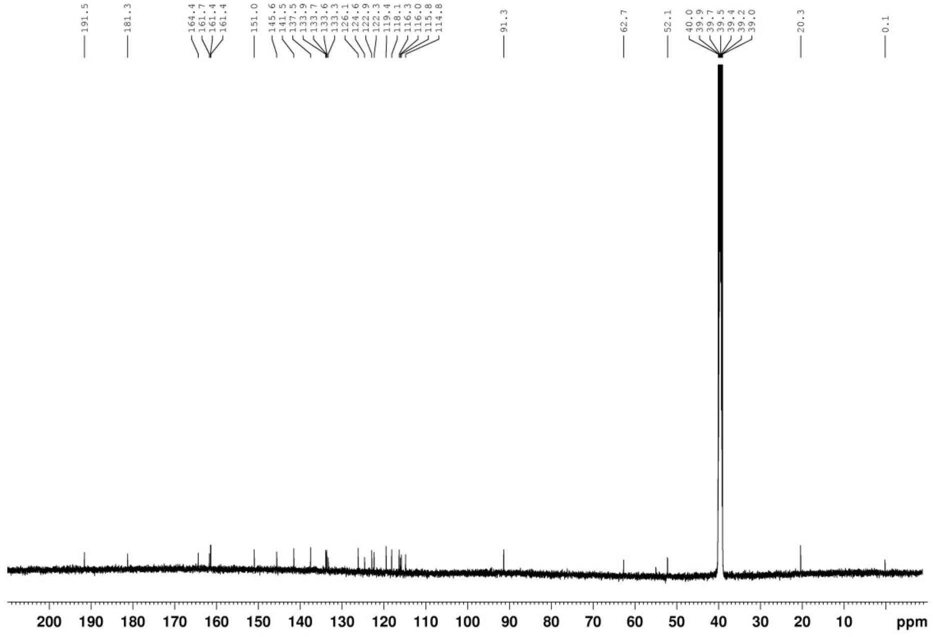

^{13}C-NMR Spectrum for 5c (DMSO-*d*6, 125 MHz).

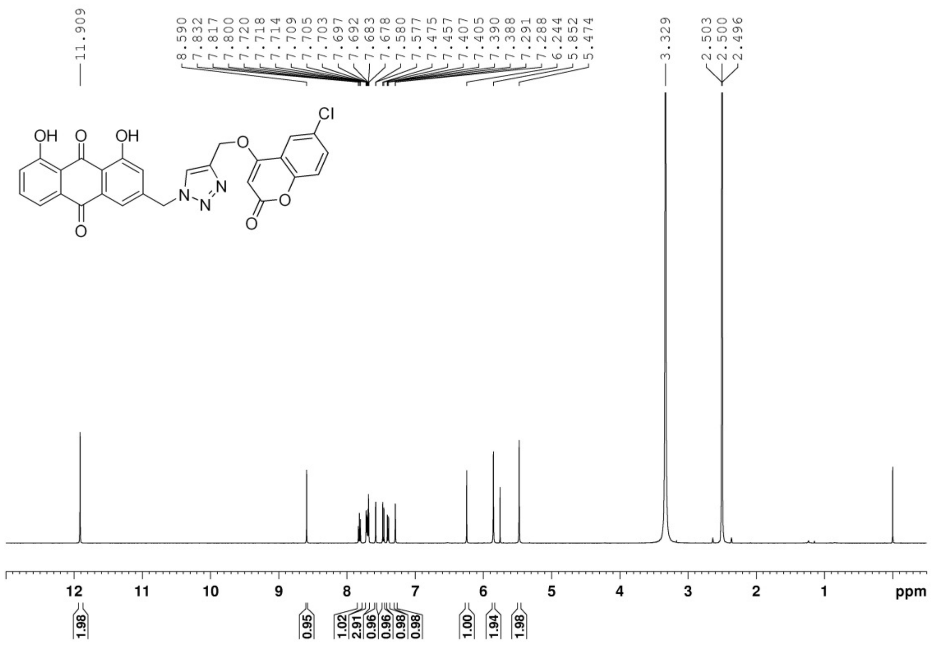

^{1}H-NMR Spectrum for 5d (DMSO-d_6, 500 MHz).

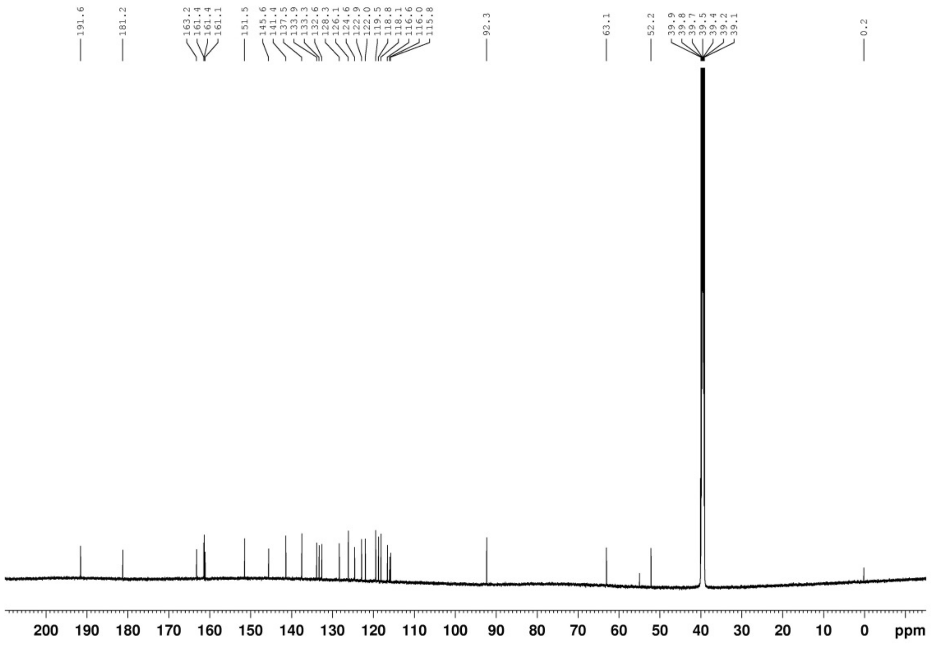

^{13}C-NMR Spectrum for 5d (DMSO-d_6, 150 MHz).

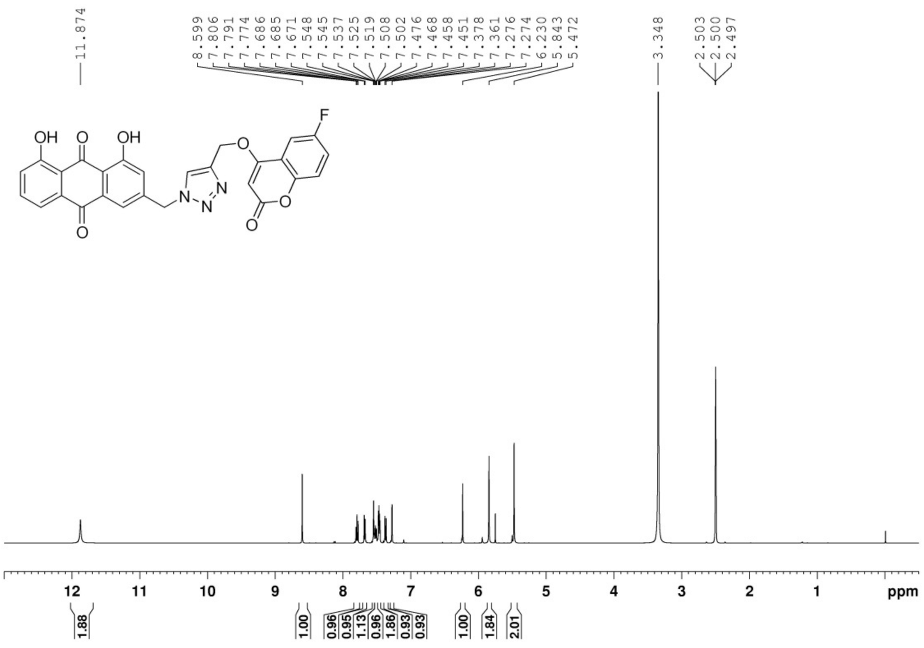

^{1}H-NMR Spectrum for 5e (DMSO-d_6, 500 MHz).

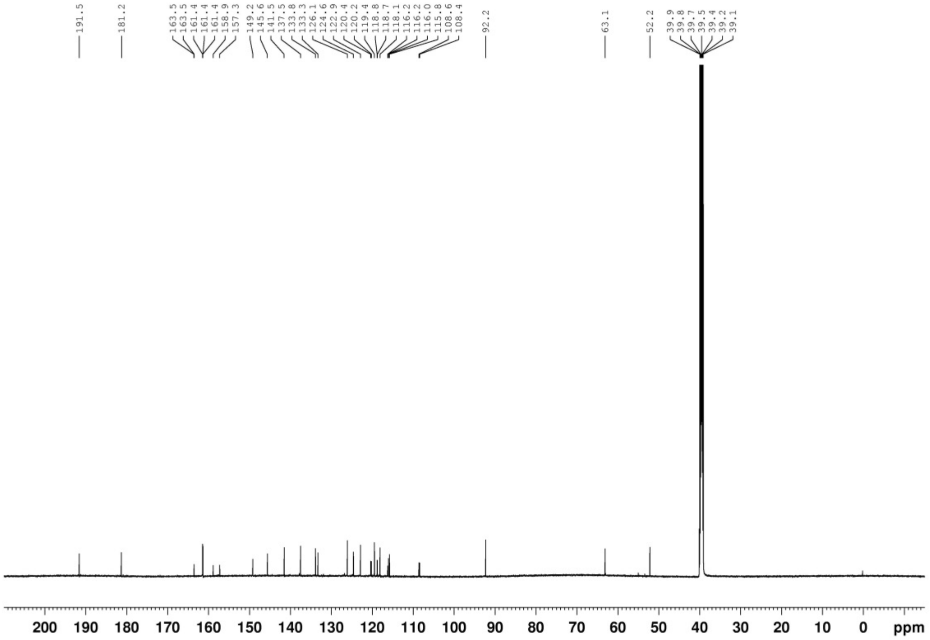

^{13}C-NMR Spectrum for 5e (DMSO-d_6, 150 MHz).

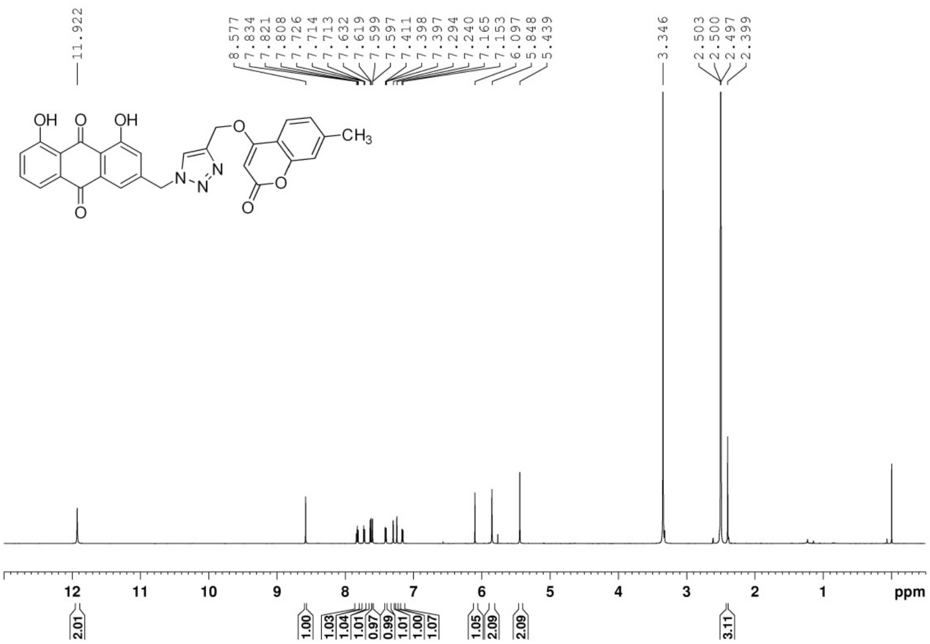

^{1}H-NMR Spectrum for 5f (DMSO-*d*$_6$, 600 MHz).

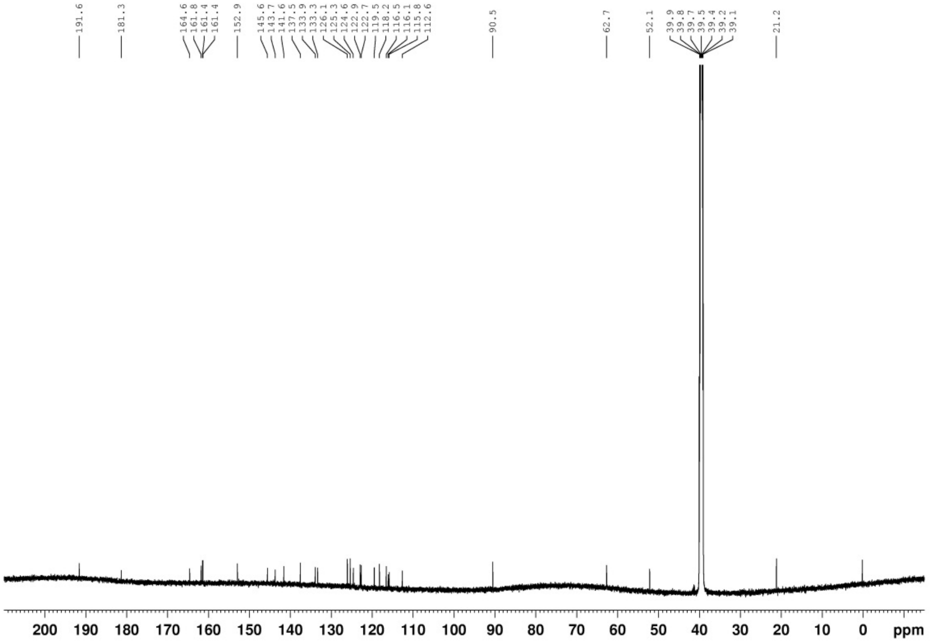

^{13}C-NMR Spectrum for 5f (DMSO-*d*$_6$, 150 MHz).

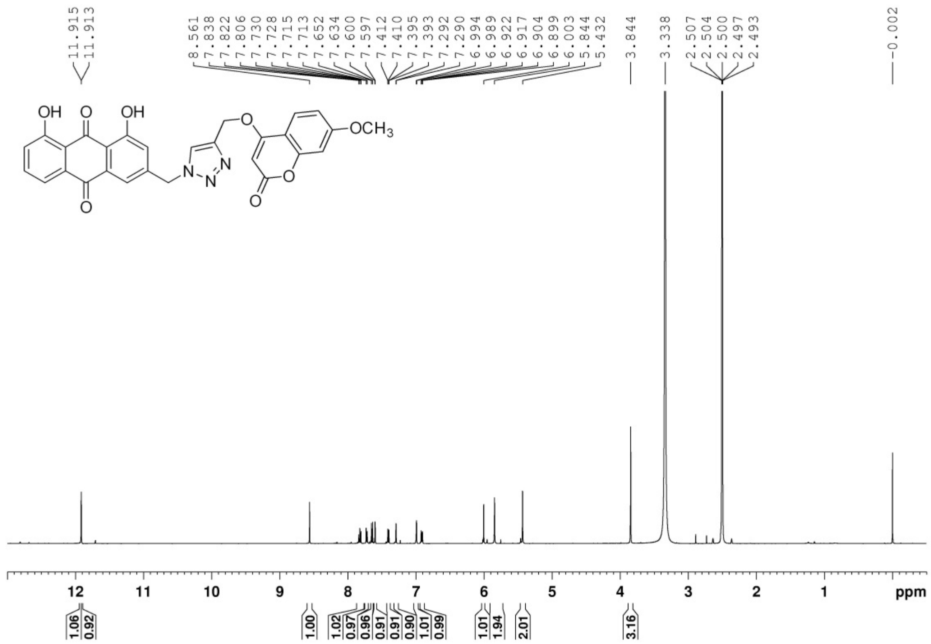

¹H-NMR Spectrum for 5g (DMSO-d_6, 500 MHz).

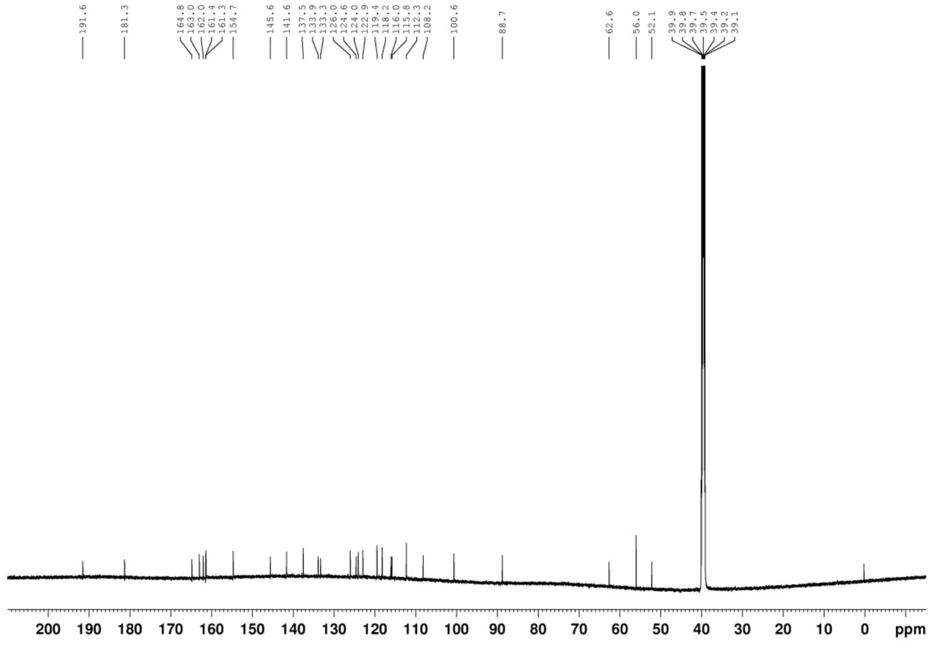

¹³C-NMR Spectrum for 5g (DMSO-d_6, 150 MHz).

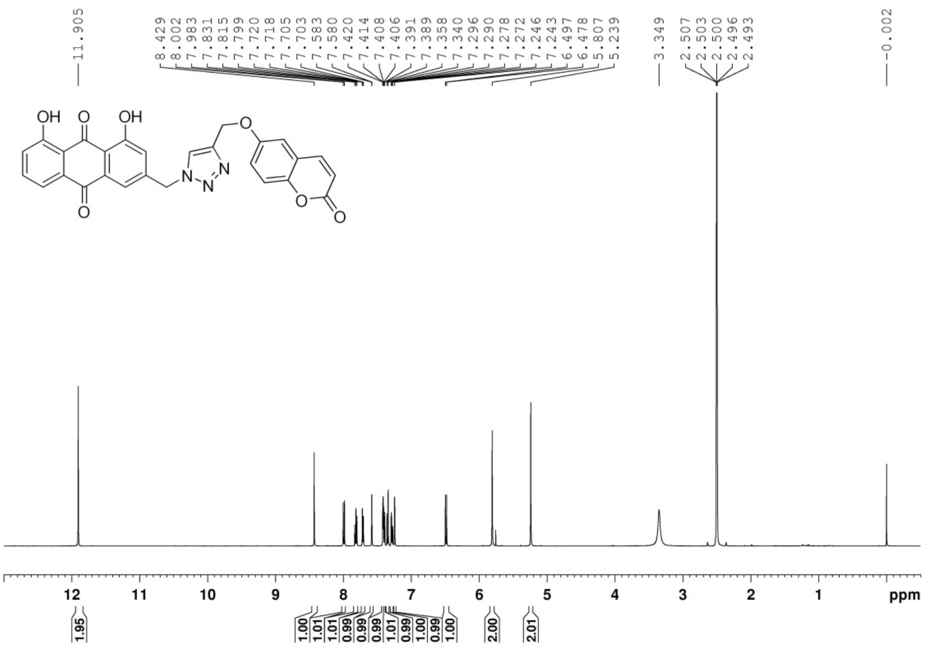

^{1}H-NMR Spectrum for 5h (DMSO-d_6, 500 MHz).

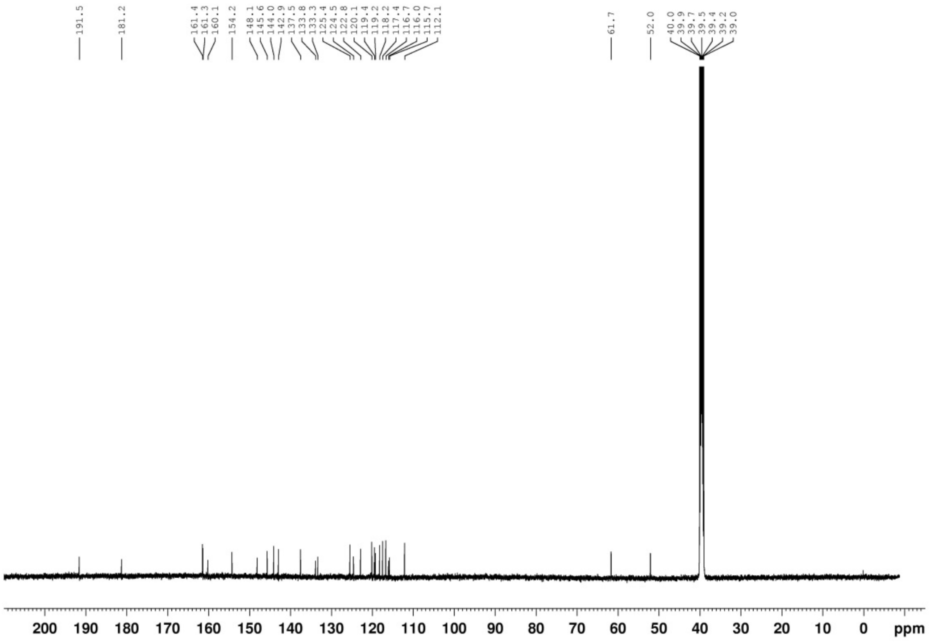

^{13}C-NMR Spectrum for 5h (DMSO-d_6, 125 MHz).

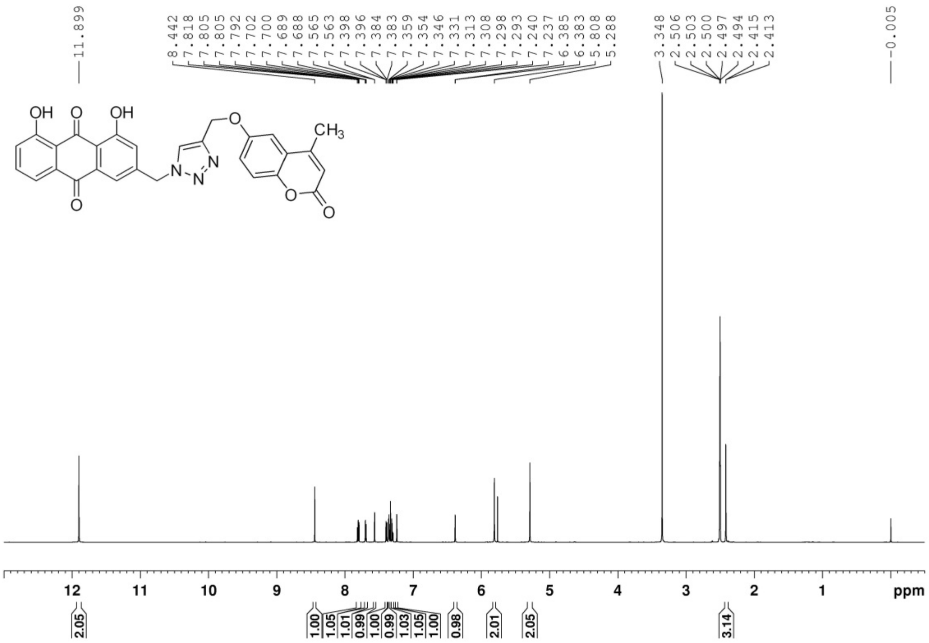

^{1}H-NMR Spectrum for 5i (DMSO-d_6, 600 MHz).

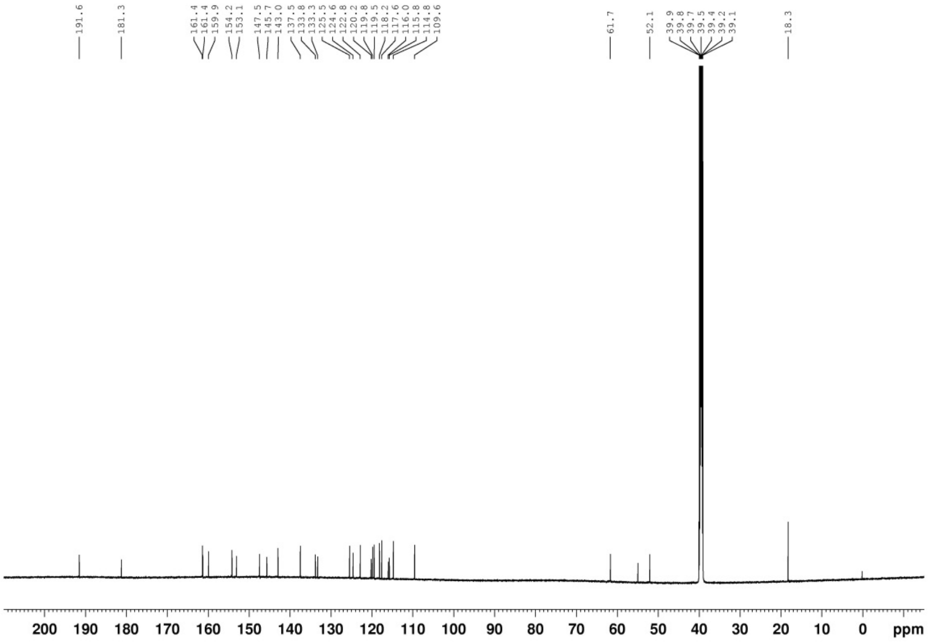

^{13}C-NMR Spectrum for 5i (DMSO-d_6, 150 MHz).

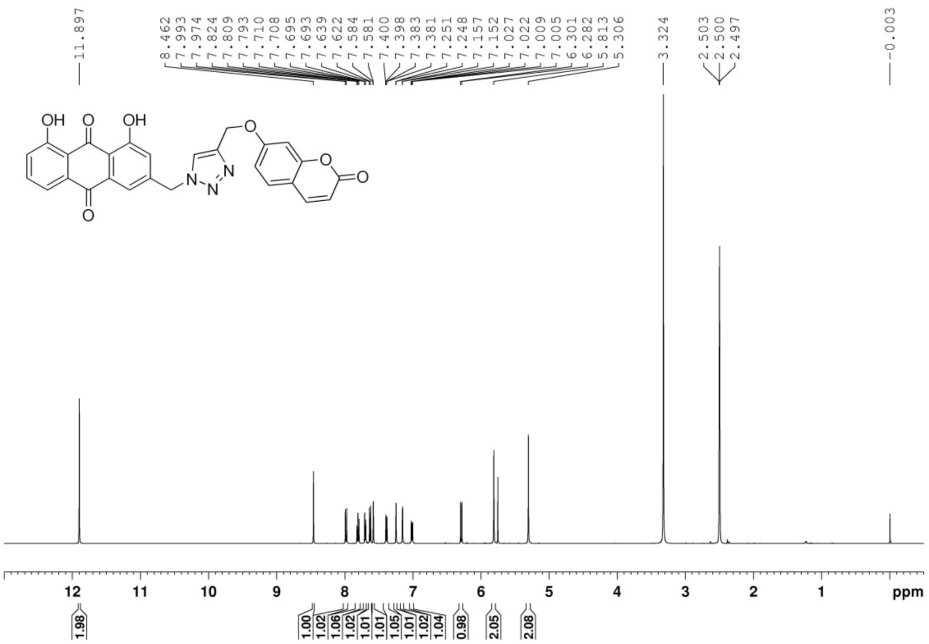

^{1}H-NMR Spectrum for 5j (DMSO-d_6, 500 MHz).

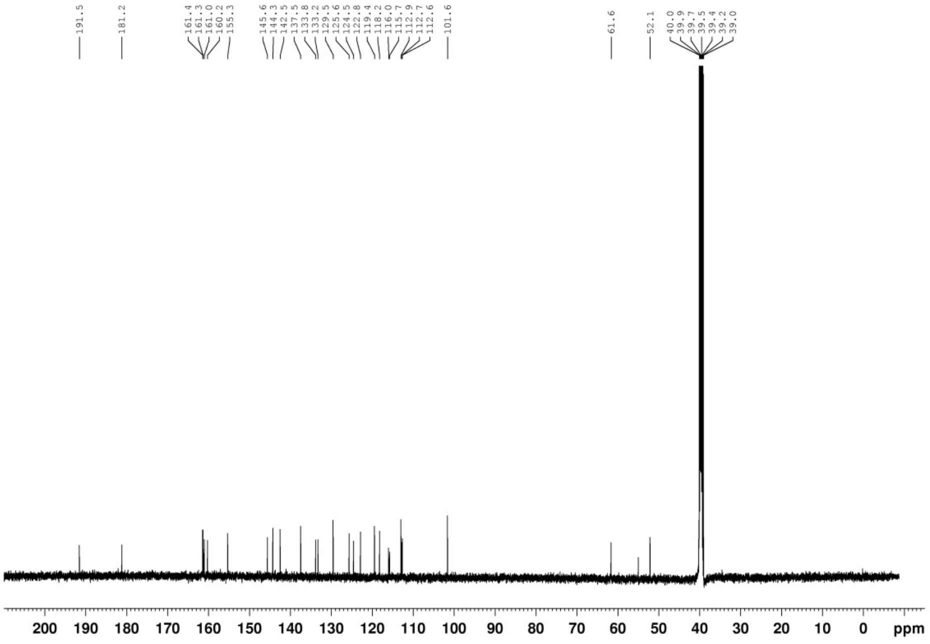

^{13}C-NMR Spectrum for 5j (DMSO-d_6, 125 MHz).

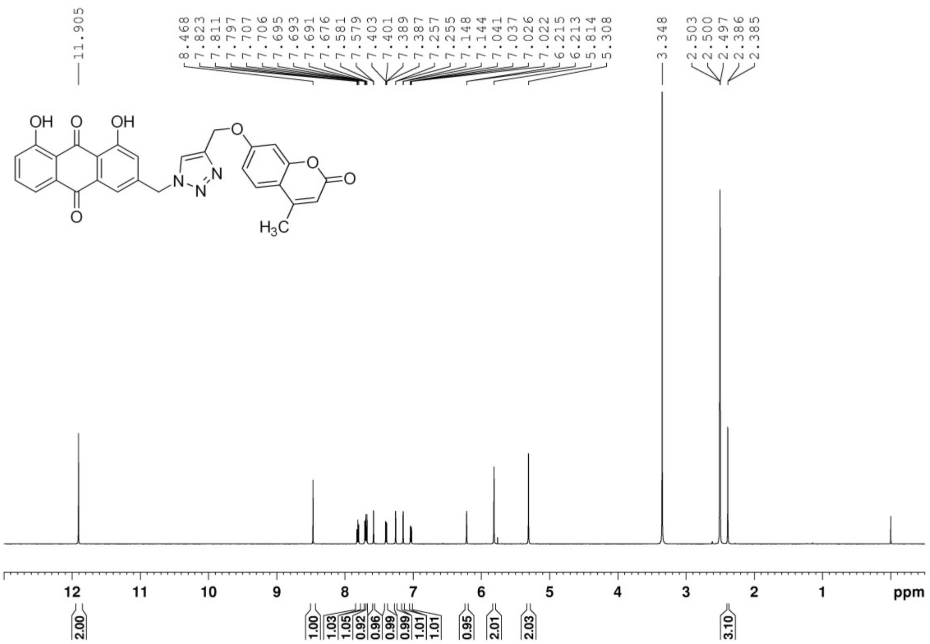

^{1}H-NMR Spectrum for 5k (DMSO-d_6, 600 MHz).

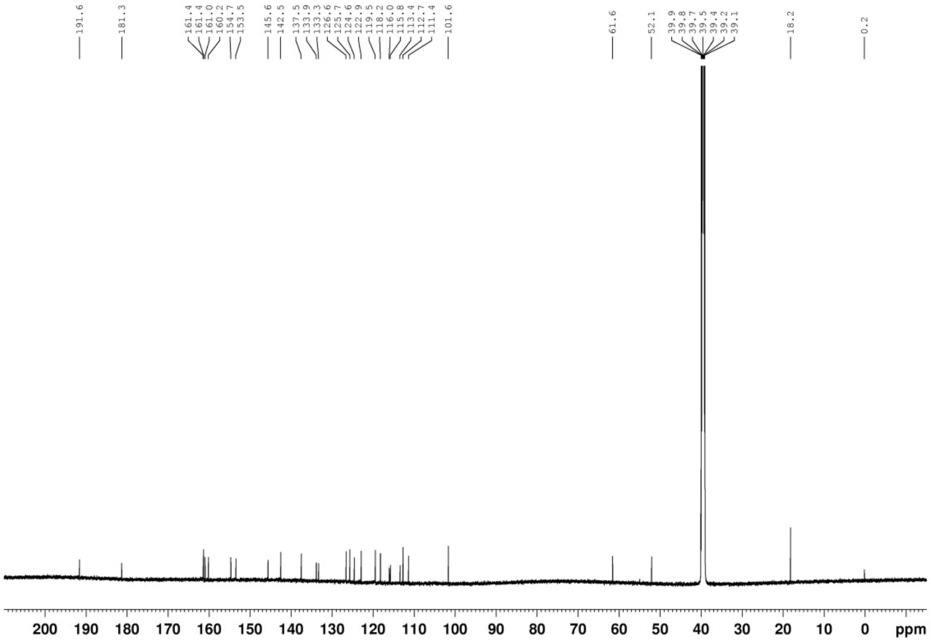

^{13}C-NMR Spectrum for 5k (DMSO-d_6, 150 MHz).

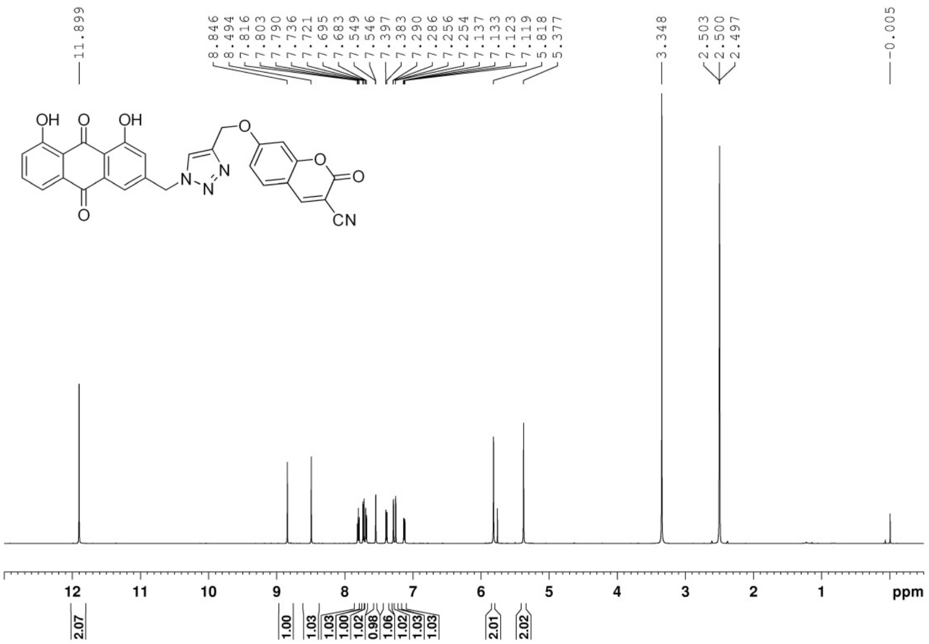

^{1}H-NMR Spectrum for 5l (DMSO-*d*$_6$, 600 MHz).

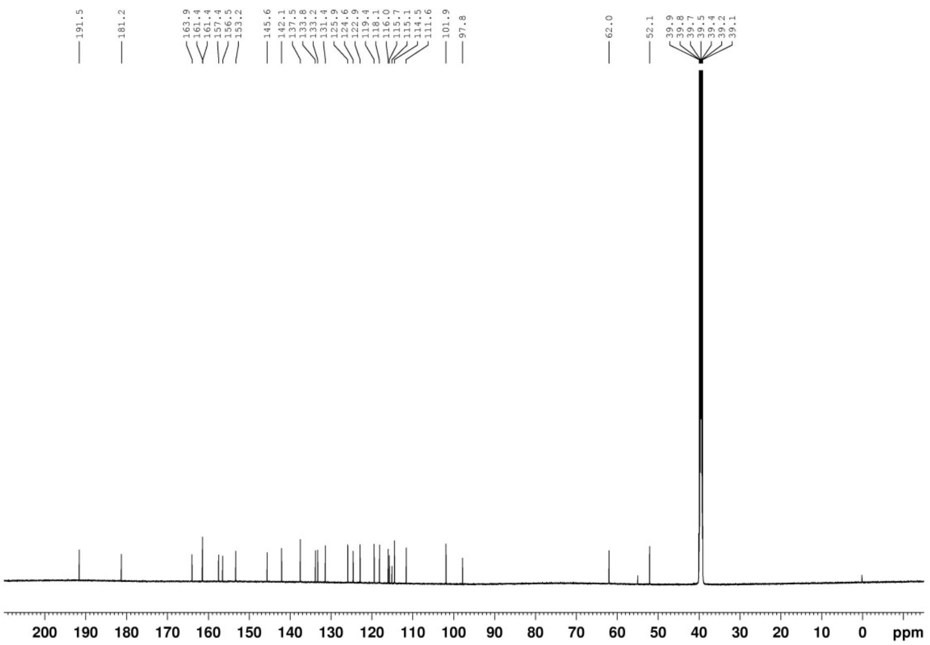

^{13}C-NMR Spectrum for 5l (DMSO-*d*$_6$, 150 MHz).

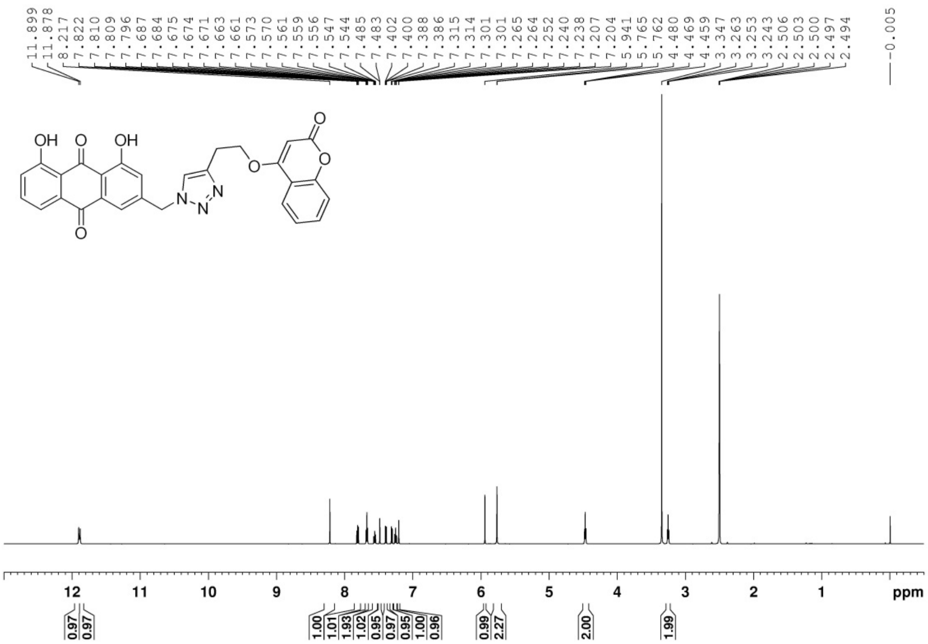

^{1}H-NMR Spectrum for 5m (DMSO-d_6, 600 MHz).

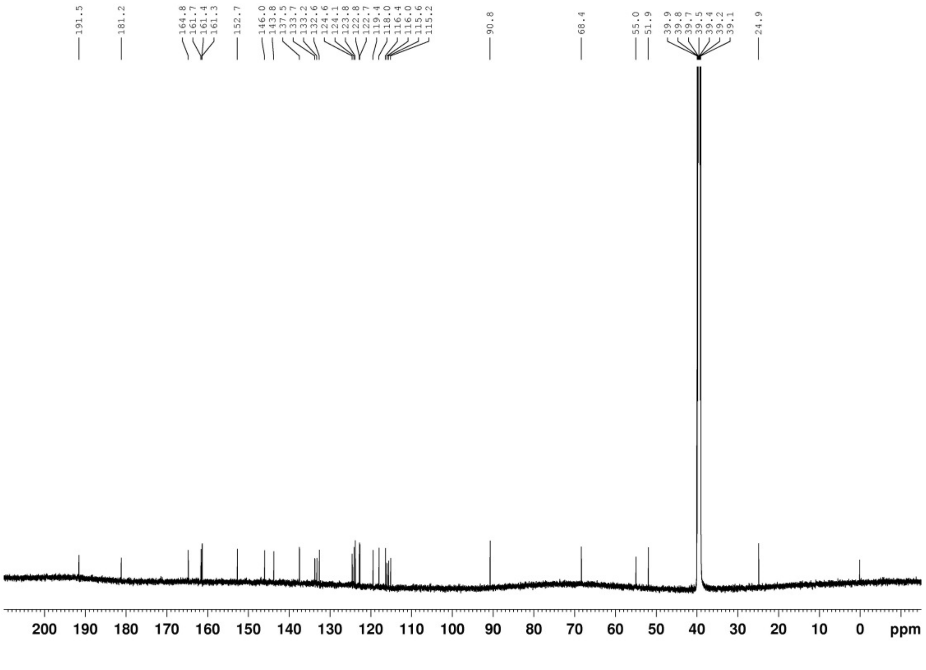

^{13}C-NMR Spectrum for 5m (DMSO-d_6, 150 MHz).

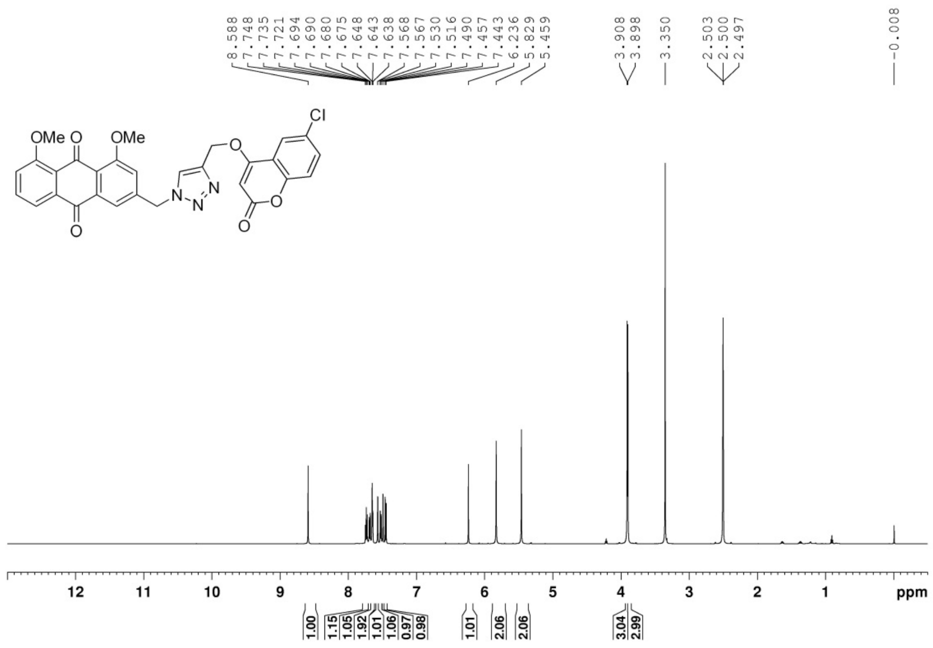

^{1}H-NMR Spectrum for 7a (DMSO-d_6, 600 MHz).

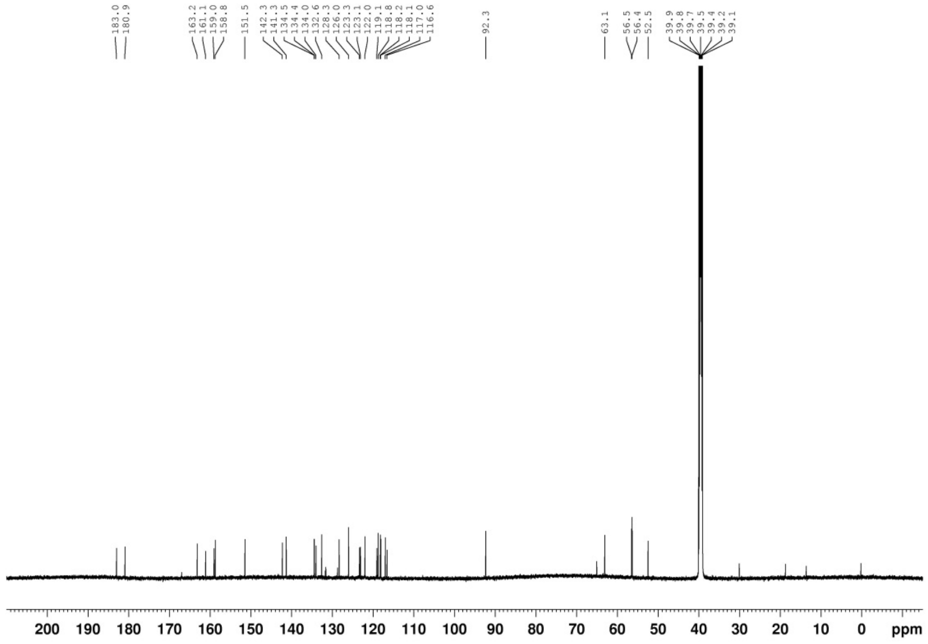

^{13}C-NMR Spectrum for 7a (DMSO-d_6, 150 MHz).

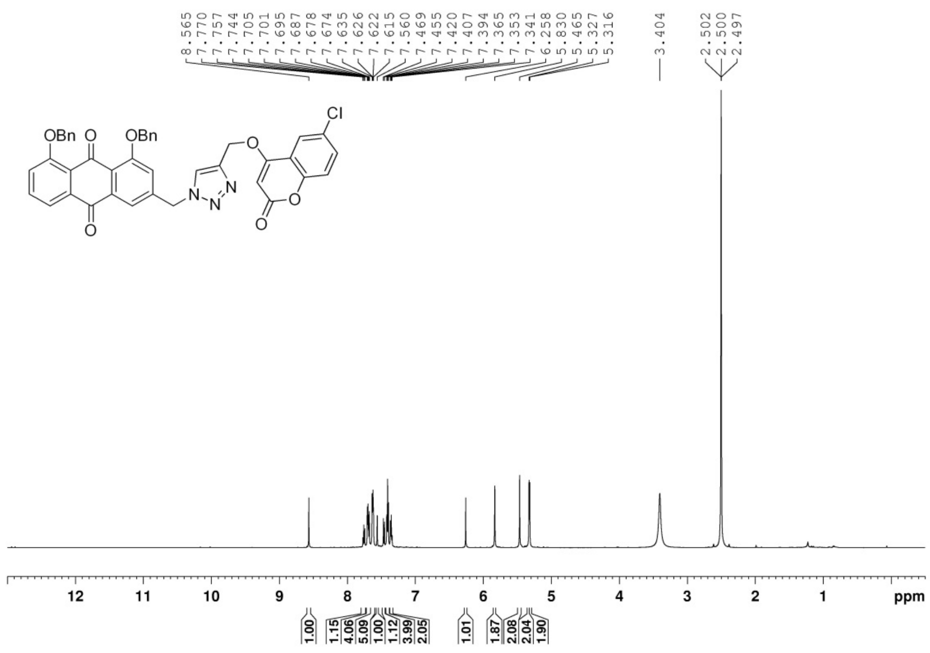

^{1}H-NMR Spectrum for 7b (DMSO-d_6, 600 MHz).

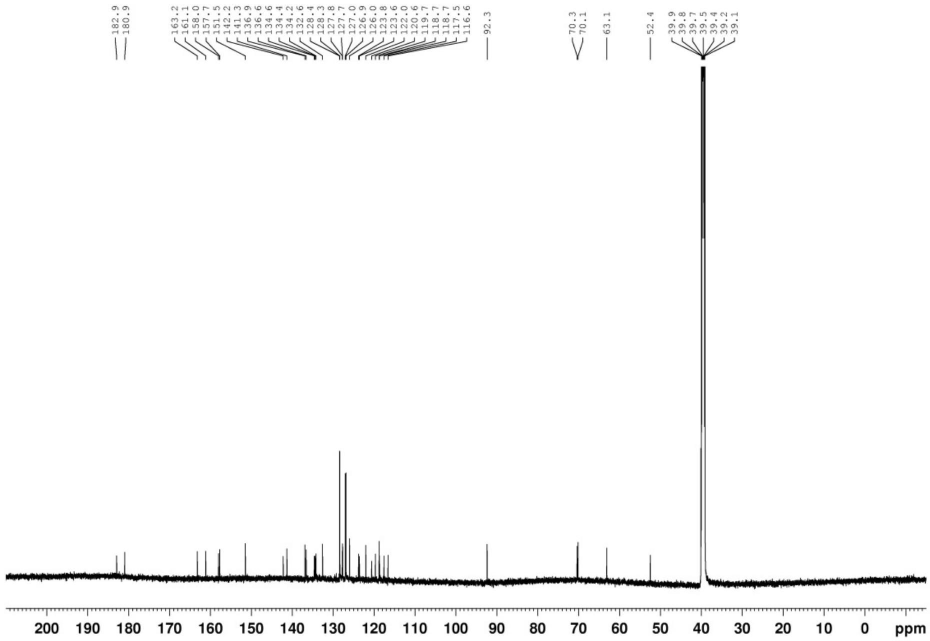

^{13}C-NMR Spectrum for 7b (DMSO-d_6, 150 MHz).

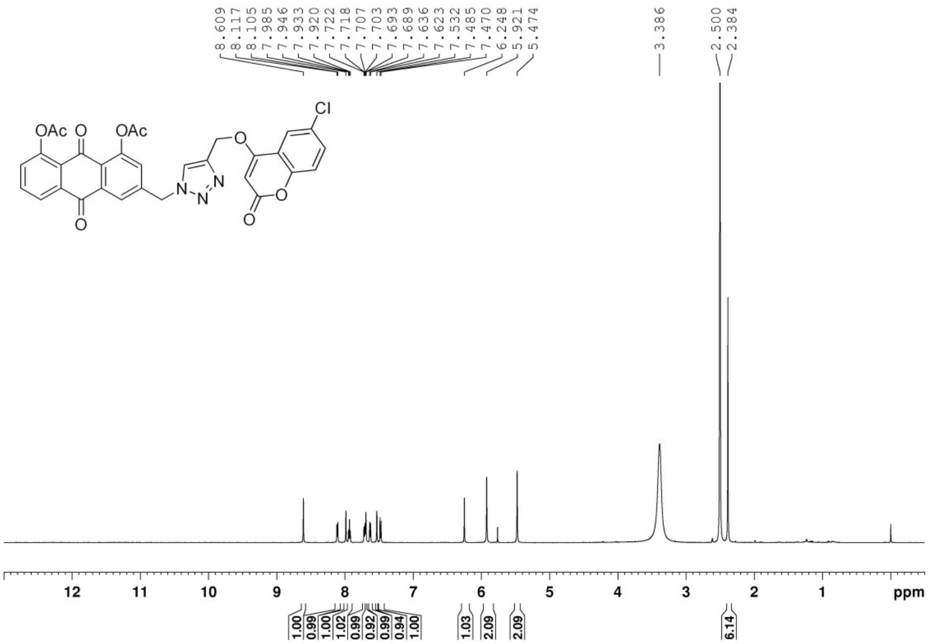

^{1}H-NMR Spectrum for 7c (DMSO-d_6, 600 MHz).

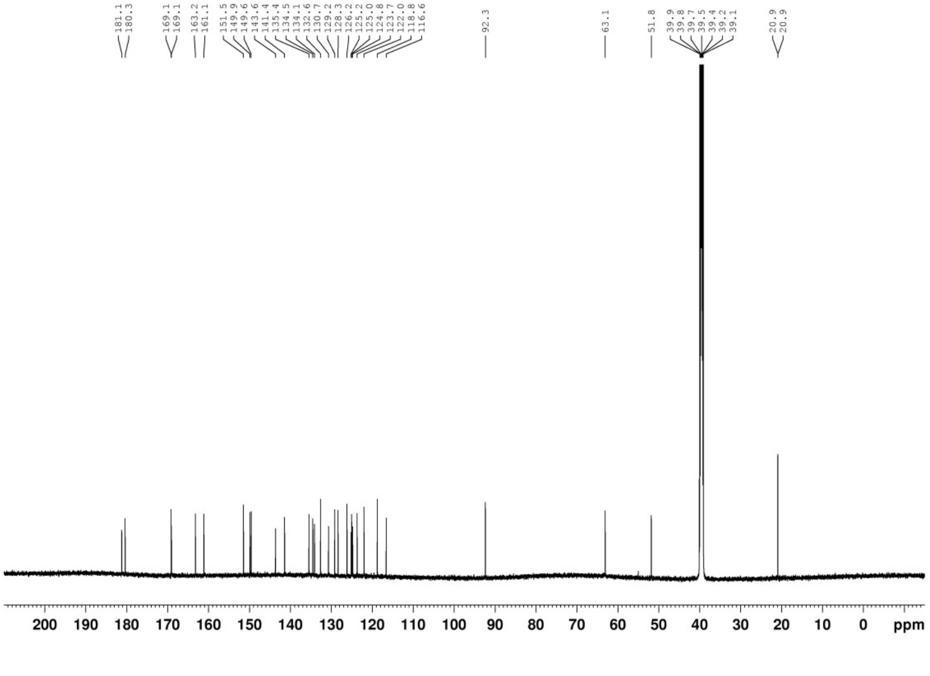

^{13}C-NMR Spectrum for 7c (DMSO-d_6, 150 MHz).

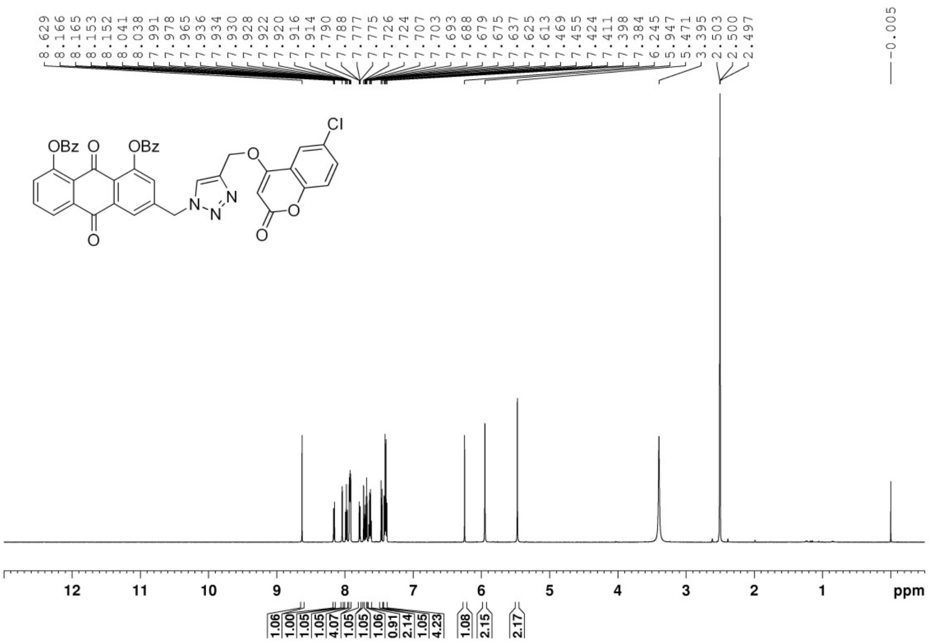

^{1}H-NMR Spectrum for 7d (DMSO-d_6, 600 MHz).

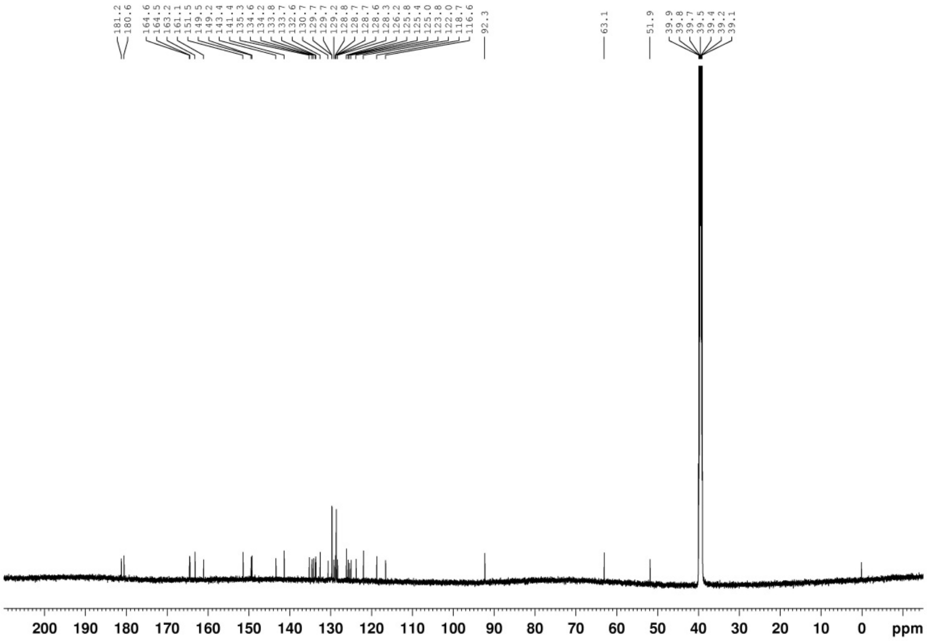

^{13}C-NMR Spectrum for 7d (DMSO-d_6, 150 MHz).

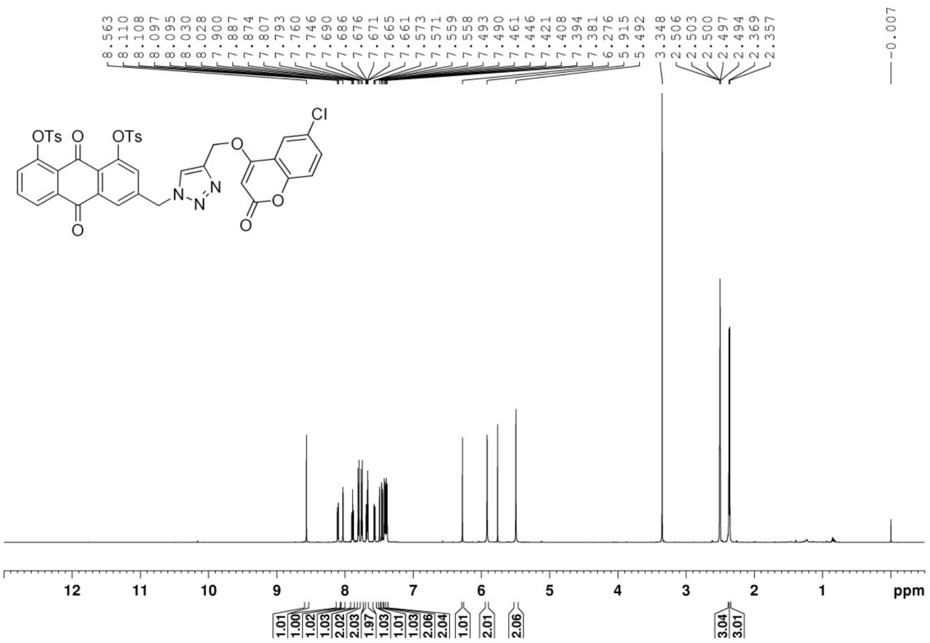

^{1}H-NMR Spectrum for 7e (DMSO-d_6, 600 MHz).

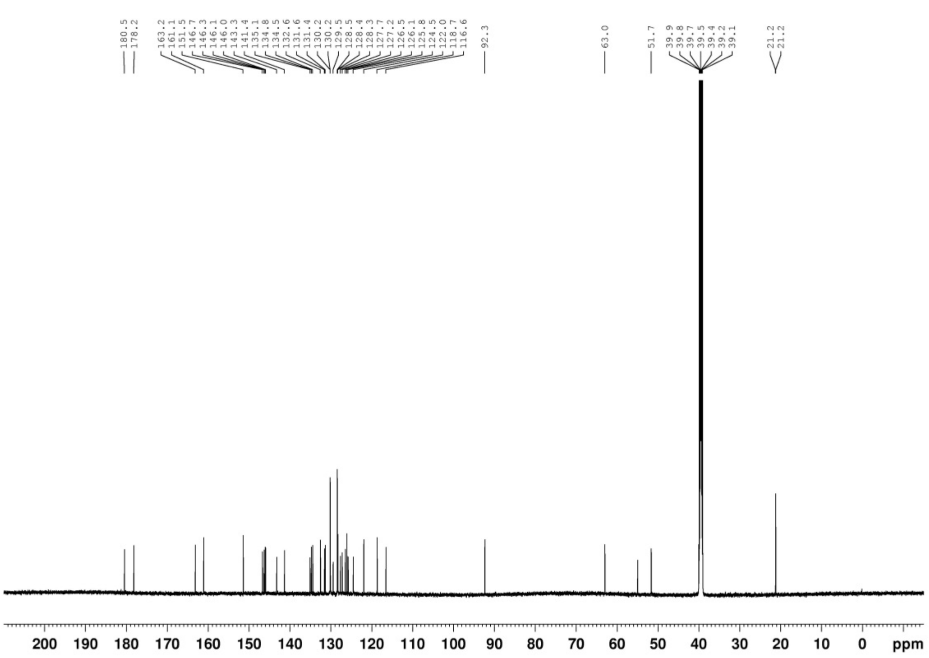

^{13}C-NMR Spectrum for 7e (DMSO-d_6, 150 MHz).
